# Clinical characteristics and drug utilisation patterns in patients with chronic cough: a retrospective cohort study using a Japanese claims database

**DOI:** 10.1186/s12890-022-02180-y

**Published:** 2022-11-21

**Authors:** Yoko Arai, Kotoba Okuyama, Yoshie Onishi, Jonathan Schelfhout, Shigeru Tokita, Takekazu Kubo

**Affiliations:** 1 MSD K.K, 1-13-12 Kudan-kita, Chiyoda-ku, Tokyo, 102-8667 Japan; 2 Creativ-Ceutical, K.K, Tokyo, Japan; 3 Merck & Co., Inc., Rahway, NJ 07065 USA

**Keywords:** Cough, Chronic cough, Central antitussives, Cough-variant asthma, Atopic cough, Allergic rhinitis, Nasal inflammation, Asthma, GERD

## Abstract

**Background:**

Although unmet medical needs for better care of patients with chronic cough exist in Japan, epidemiological information about these patients and their treatments is very limited.

**Objectives:**

To describe patient characteristics, underlying cough-related diseases and drug utilisation patterns in patients with chronic cough, and their changes over time.

**Methods:**

This large retrospective claims database study enrolled subjects with chronic cough, identified either by a specific diagnostic cough code for chronic cough (Population 1) or by multiple cough-related diagnostic codes spanning > 8 weeks (Population 2). Within Population 2, patients with each of the three most frequent diagnostic cough codes were analysed as subgroups. Patient characteristics, underlying cough-related diseases and utilisation patterns for drugs used for cough were documented at the index date, during the 6-month pre-index period and during the 12-month post-index period.

**Results:**

6,038 subjects were enrolled in the cohort (Population 1: *N* = 3,500; Population 2: *N* = 2,538). The mean age was 43.7 ± 12.2 years and 61.8% were women. The largest cough diagnosis subgroups in Population 2 were ‘other coughs’ (*N* = 1,444), ‘cough-variant asthma’ (*N* = 1,026) and ‘atopic/allergic cough’ (*N* = 105). At the index date, the most frequent underlying cough-related diseases were allergic rhinitis/nasal inflammation (*N* = 3,132; 51.9%), asthma (*N* = 2,517; 41.7%) and gastro-esophageal reflux disease (*N* = 829; 13.7%). At the index date, 4,860 participants (80.5%) were prescribed at least one cough-related treatment. 194 participants (4.0% of medication users) were prescribed central antitussives alone, principally in Population 1, and 2,331 (48.0%) were prescribed expectorants. Other frequently prescribed medications were antiallergic drugs (*N* = 2,588; 53.3%), antimicrobials (*N* = 1,627; 34.4%) and inhaled corticosteroids with long-acting beta-agonists (*N* = 1,404; 28.9%). Over time, cough diagnoses tended to be lost, with only 470 participants in Population 1 retaining a diagnostic code for chronic cough one year later. The frequency of underlying cough-related diseases was stable over time.

**Conclusions:**

Patients in this cohort with chronic cough are most frequently identified by a diagnostic cough code for chronic cough, followed by codes for other coughs, cough-variant asthma and atopic cough. Chronic cough frequently presents with an underlying cough-related disease, most frequently allergic rhinitis/nasal inflammation, asthma or GERD. Medication prescription for the underlying cough-related diseases was generally appropriate.

**Supplementary Information:**

The online version contains supplementary material available at 10.1186/s12890-022-02180-y.

## Introduction

Cough is one of the most frequent reasons for which patients seek help from primary care physicians. Acute cough is commonly caused by upper respiratory tract infections and typically lasts no more than three weeks. However, certain patients present with persistent chronic cough, defined as a cough lasting more than 8 weeks [1], which is associated with impaired quality of life and a significant disease burden in terms of psychological and physical impact [2–4]. Chronic cough may be due to post-infectious complications or present as a symptom of an underlying chronic disease such as asthma, allergic disorders of the upper respiratory tract or gastro-esophageal reflux disease (GERD) [5, 6]. However, the aetiology is frequently unidentified or may involve multiple components [5, 7]. In addition, it has been suggested that chronic cough may not just be a symptom of underlying disease, but rather a disease entity in itself [8–10]. According to this hypothesis, chronic cough would be caused by a hypersensitivity syndrome in which the cough threshold to thermal, mechanical and chemical stimulation is lowered [8–10].

Epidemiological studies of chronic cough have yielded a range of prevalence rates ranging from 1 to 12%, [11–16], with the rate in the general adult population likely to be in the range of 4–5% [16–18]. Prevalence is higher in older individuals and in women [11, 16, 17]. In Japan, the point prevalence of cough (regardless of duration) in the general population has been estimated to be 10.2%, with no difference in prevalence between men and women or between age groups [12]. However, for chronic cough lasting ≥ 8 weeks, the prevalence was around 2%, being somewhat higher in men (2.5%) than in women (1.4%), and tending to rise with age [12].

Guidelines for the diagnosis and management of chronic cough have been developed by professional bodies in several countries including Japan [5, 6, 19]. The guidelines of the Japanese Respiratory Society (JRS) provide a comprehensive algorithm for the initial assessment, diagnosis, classification and treatment of chronic cough in adults [19]. The Japanese guidelines emphasise the importance of performing an adequate diagnostic work-up in all patients with a cough lasting more than three weeks, and then treating any underlying diseases appropriately [19]. These guidelines recommend treatment of productive cough consistent with sinobronchial syndrome with erythromycin or a related antibiotic [19]. Dry cough with suspected allergic or atopic origin should be treated with antihistamines or inhaled corticosteroids (ICS), treatment of cough-variant asthma with long-acting beta-agonists (LABA) and ICS and cough associated with GERD with anti-reflux treatments [19]. Mucolytics (expectorants) are recommended for the management of productive cough [19]. Centrally acting (narcotic and non-narcotic) antitussives are not recommended in the Japanese guidelines due to adverse effects such as constipation and drowsiness, and due to nonspecific cough suppression which may mask the evaluation of the treatment response for any underlying diseases [19] In addition, these medications suppress “necessary coughing” that acts as a physiological defence mechanism to eliminate foreign substances or pathogens invading the airways [5, 6, 19]. Finally, in many cases these medications are ineffective [19]. Despite a range of recommended treatments for chronic cough management, treatment satisfaction in Japan is low, at around 16.0% [15]. Approximately 40% of surveyed individuals with chronic cough were receiving medical care for their cough, and only half of these were under care for longer than 1 month [12].

In Japan, little information is available on patterns of treatment for chronic cough, and notably with respect to the description of underlying diseases and antitussive use. To the best of our knowledge, no study using real-world data in the Japanese population has evaluated chronic cough management. Health insurance claims databases represent a powerful tool for collecting comprehensive data on comorbidities and healthcare resource consumption in identified populations with specific diseases. For this reason, we have performed a retrospective cohort study using a large Japanese health insurance claims database in order to describe patients with chronic cough and their management in the Japanese setting for the first time.

The objective of the present study was to describe the characteristics of adult patients with chronic cough in Japan, in terms of demographic and clinical characteristics, common underlying cough-related diseases and cough-related drug utilisation patterns. An exploratory objective was to estimate the number of adults with chronic cough in Japan.

## Methods

In this retrospective cohort study, data were extracted from a Japanese health insurance claims database (Medi-Scope®), provided by Japan Medical Information Research Institute Inc. (JMIRI). Patient records covering the period from 1st January 2017 until 31st August 2020 were included. Demographic and clinical characteristics of adult patients with chronic cough were described cross-sectionally and clinical outcomes and drug utilisation patterns were evaluated longitudinally.

### Data source

Data were derived from the Medi-Scope® database. This administrative claims database, established in 2010, is one of the largest in Japan, compiling reimbursement claims from over seventy health insurance associations and covering approximately 6.7 million beneficiaries (around 5.2% of the entire Japanese population). Beneficiaries are employees or their family members under 75 years of age. Medi-Scope® provides claims data for inpatient, outpatient, dental and pharmacy claims arising from both the public and private sector. It should be noted that patients have a free access to medication institution regardless of specialities and/or affiliations of physicians in the Japanese healthcare system, therefore, there is no clear distinction between primary and secondary care in Japan as all individuals in need of medical advice consult at a hospital or a clinic. Diagnoses are documented by the appropriate International Classification of Diseases 10th Edition (ICD-10) code. A special feature of the Japanese database is that, as well as the ICD-10 code, diseases are also identified by a standard disease name in the Japanese vernacular, which may be more specific than the ICD-10 code. For example, the single ICD-10 code for cough (R05) is subdivided into a range of different cough types with different Japanese vernacular names. Each beneficiary is assigned a unique identifier, which allows their healthcare resource utilisation to be followed over time. Demographic data include gender, year and month of birth, and whether the beneficiary is an employee or family member. Prescription claims are documented on the day, month and year the prescription was filled. On the other hand, consultations are only documented on a monthly basis in Japan, and for this reason, information on ICD-10 diagnoses can only be identified by the month and year of diagnosis and it is not possible to capture the diagnosis date with greater precision. In the present study, the index date is given as index month and prescription date is given as the actual date of prescription. In addition, if a beneficiary leaves their insurance scheme for any reason, this information is not available.

### Definition of populations of interest

The study was restricted to participants aged ≥ 20 years. Two populations of interest were identified (Populations 1 and 2), characterised by the presence of different diagnostic cough codes. Any participants who were eligible for both Population 1 and Population 2 were affected to Population 1. In this way, the two populations were mutually exclusive. Population 1 consisted of those participants with an ICD-10 diagnostic code for cough (R05) associated with the Japanese standard vernacular name for chronic cough documented in the database at least once.

Since it was anticipated that participants fulfilling criteria for chronic cough may be coded in the database with other types of cough code [15], a second population (Population 2) was selected, defined by the presence of other ICD-10 diagnostic cough codes and Japanese specific disease names. These cough codes are listed in Table 1. The choice of diagnostic cough codes (Table 1) was based on the most frequent types of coughs identified in an earlier observational study conducted to elucidate the causes of chronic cough in Japan [20], and identified in the JRS practice guidelines [19].

**Table 1:** Diagnostic cough codes and standard disease names used to identify Populations 1 and 2

Cough category name	ICD-10 code	ICD-10 description	Standard disease name
*Population 1*			
Chronic cough	R05	Cough	Chronic cough
*Population 2*			
Cough associated with infection	A16.9	Respiratory tuberculosis unspecified, without mention of bacterial or histological confirmation	Tuberculous cough
	A37.0	Whooping cough due to *Bordetella pertussis*	Whooping cough caused by *B. pertussis*
	A37.9	Whooping cough unspecified	Whooping cough
Atopic / allergic cough	R05	Cough	Atopic cough
	R05	Cough	Allergic cough
Cough-variant asthma	J45.9	Asthma, unspecified	Cough-variant asthma
Postinfectious cough	R05	Cough	Post-infectious cough
Other coughs	F45.3	Somatoform autonomic dysfunction	Psychogenic cough
	G44.8	Other specified headache symptoms	Primary cough headache
	R05	Cough	Catarrhal cough
	R05	Cough	Cough
	R05	Cough	Syncope cough
	R05	Cough	Dry cough
	R05	Cough	Wet cough
	R05	Cough	Prolonged subacute cough
	R05	Cough	Nocturnal cough

*ICD-10* International classification of diseases – 10th edition

Eligibility for Population 2 required documentation of at least two diagnostic cough codes in two different months within an evaluation period of three consecutive months, followed by at least one other diagnostic cough code in the subsequent three-month post-evaluation window. Documentation of multiple consultations for cough was required in order to ensure, in the absence of information on the specific dates of the consultations for cough, that individuals in Population 2 presented a cough lasting more than eight weeks and thus fulfilled the accepted duration criterion for chronic cough [1].

Within Population 2, cough types were grouped into five categories (cough associated with infection, atopic/allergic cough, cough-variant asthma, postinfectious cough and other coughs). If more than one category of diagnostic cough code was documented during the evaluation period and the post-evaluation window, then the participant would be assigned to both cough subtype groups, and these subgroups are thus not mutually exclusive.

### Study timelines, periods and dates

**Fig. 1:**
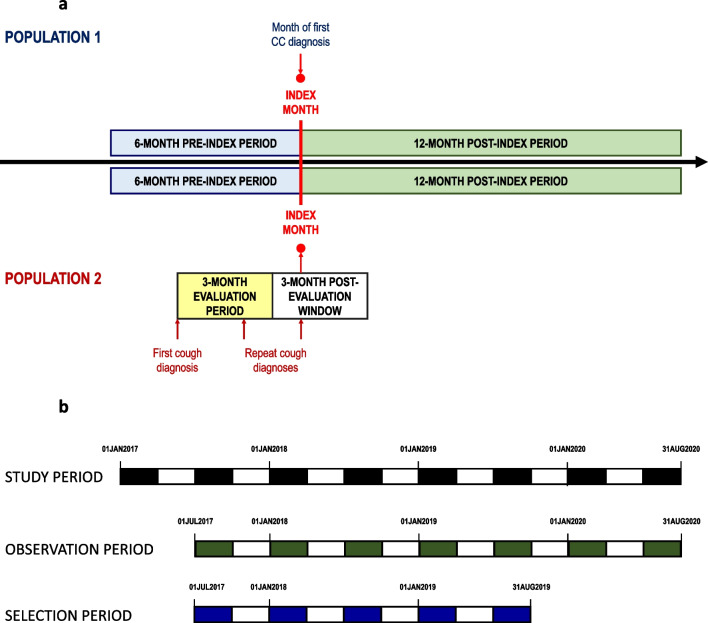
Study Design **a** Study populations. The index month corresponds to the first month during the selection period in which a chronic cough diagnosis was confirmed for each individual patient. In Population 1, this corresponds to the first documentation of a diagnostic code for chronic cough. In Population 2, this corresponds to the first new cough diagnosis observed within a 3-month window following any 3 month period during which at least two diagnostic cough codes were documented in two different months. The index month was preceded by a 6-month pre-index period, during which historical information on cough-related diagnoses was retrieved. The index month was followed by a twelve-month post-index period, during which information on treatments and cough and cough-related diagnoses were collected**.** CC: Chronic cough. **b **Study timelines, periods and dates. The selection period (blue and white bars; 1st July 2017 to 31st August 2019) corresponds to the period when patients could be included. The observation period (green and white bars; 1st July 2017 to 31st August 2020) corresponds to the selection period, together with the 12-month post-index period for each patient. The study period (black and white bars; 1st January 2017 to 31st August 2020) corresponds to the observation period, together with the 6-month pre-index period for each patient

Study timelines, periods and dates are presented in Fig. 1. Participants were enrolled retrospectively over a 26-month selection period between 1st July 2017 and 31st August 2019.

The index month was defined as the month in which the diagnosis of chronic cough was first established. In Population 1, this was defined as the first month in which the relevant diagnostic cough code was documented in the Medi-Scope® database during the selection period. In Population 2, the index month was defined as the month in which the relevant diagnostic cough code was first documented in the database during the 3-month post-evaluation window following the end of the 3-month evaluation period. For Population 2, it should be noted that the diagnostic cough code identified in the index month may be different to that documented in the evaluation period, since, in this population, all relevant diagnostic cough codes will be captured throughout the evaluation period and the post-evaluation window.

In addition, a data extraction period of 6 months immediately preceding the index month was identified (pre-index period), as well as an observation period of 12 months immediately following the index month (post-index period). The total observation period began on 1st July 2017 and ended on 31st August 2020 to allow a sufficient follow-up for the analysis of drug utilisation. The total study period thus lasted from 1st January 2017 until 31st August 2020.

### Other eligibility criteria

Participants with a prescription of angiotensin-converting enzyme inhibitors, identified by Anatomical Therapeutic Chemical (ATC) class codes C9A or C9B, at any time during the study period were excluded, as were participants with organic respiratory diseases (such as interstitial lung disease, cystic fibrosis or pulmonary fibrosis) or with any form of cancer. The diagnostic codes used to identify these diseases are provided in Additional file 1: Material Table 1.

Participants were required to have been documented in the database for least six months prior to the index month (pre-index period) and to have at least one medical or prescription reimbursement claim during the 12 months following the index month (post-index period). Patients for whom this was not the case were excluded.

### Variables of interest extracted from the Medi-scope® database

At the index month, information on the age and gender of participants and the size of the medical faculty where they were treated was extracted from the database. Since chronic cough can persist for many years despite appropriate treatments for underlying diseases [10], we investigated changes over time in documentation of diagnostic cough codes, underlying diseases potentially associated with the cough (henceforward called ‘cough-related diseases’) and medications potentially used for the management of chronic cough or cough-related diseases. In order to understand what types of medication could be provided to patients actively seeking treatments for cough, we determined the proportion of patients receiving cough-specific medications or medications for cough-related diseases and evaluated how these proportions varied across the post-index period.

Diagnostic cough codes were documented throughout the pre-index and post-index periods in order to evaluate their persistence over time using the same definitions as for the patient selection algorithm.

Information on cough-related diseases, such as emphysema, chronic rhinitis or GERD, were identified in the Medi-Scope® database from the relevant ICD-10 diagnostic codes. These codes are listed in Additional file 1: Table 1. The choice of cough-related disease codes was based on the most frequent types of cough-related disease/comorbidity identified in several Japanese observational studies [20, 21] and in the JRS practice guidelines [19]. These were allergic rhinitis/nasal inflammation, GERD, paranasal sinusitis, chronic airway inflammatory disease, chronic rhinitis, postnasal drip, vocal cord dysfunction, emphysema and nasal polyps. Since participants could have more than one cough-related disease, these categories were not mutually exclusive. Information on cough-related diseases was extracted for three periods, namely the index month, during the entire 6-month pre-index period (including the index month) and during Months 10 to 12 of the post-index period. A 3-month time window (Months 10–12) was considered the minimum necessary to capture all disease-specific medical claims in insurance claims databases, as previously reported [22].

Medications potentially used for the management of chronic cough or cough-related diseases were also documented. Medication classes of interest were central antitussives and expectorants (cough management medications), antiallergic agents, corticosteroids, long acting β-agonists, other bronchodilators, antimicrobials, drugs for peptic ulcer management or improving gastrointestinal transit and herbal medicines. Antimicrobials were restricted to respiratory quinolones and 14- and 15-member ring macrolides, which are recommended for their anti-inflammatory properties and sputum suppression [23] as cough treatments for adults in JRS guidelines for chronic cough management [19]. Medications of interest are listed in Additional Material file 1: Table 2. Medications were identified by ATC class from prescription records retrieved from the database for the index month and for Months 1, 2, 3, 6, 9 and 12 of the post-index period. Except for the index month, prescription records for two weeks before and after each time point (in total, four weeks) were collected and presented as the drug utilisation pattern at 1, 2, 3, 6, 9 and 12 months following the index date. In the JRS practice guidlines, evaluation of the efficacy of medication is recommended every two-weeks. A two week collection period was therefore applied according to Japanese clinical practice.

### Statistical analysis

#### Analysis populations

In this report, data are presented for the following six populations: all eligible participants enrolled with a chronic cough diagnosis (Population ‘All’), Population 1, Population 2 and the top three subgroups of Population 2 with the largest number of patients.

For the analysis of data for the index month and the pre-index period, all percentages were calculated using the total number of participants in each population or subgroup at inclusion as the denominator. For the analysis of the data on the proportion of participants with different types of cough diagnoses and cough-related diseases during Months 10 to 12 of the post-index period, the percentages were calculated using as the denominator the number of participants with either a diagnostic cough code or a cough-related disease claim documented during the month of interest. For the analysis of the data on the proportion of patients with prescription claims of interest during the index month and during the post-index period, the percentages were calculated with the number of participants with any prescription claim for a medication of interest documented during the month of interest used as the denominator. Percentages showed the proportion of patients with claims of interest during the month of interest. Change of percentage of repartition of patients at the month of interest were therefore shown in this paper.

#### Statistical methods

The analysis of the study data was purely descriptive and no statistical hypotheses were formulated or tested. For continuous variables, sample sizes (n) and mean values with their standard deviation, or median values with their interquartile range are provided. For categorical variables, frequency counts and proportions in each category of interest are provided. Missing data were not replaced. All analyses were performed using SAS® (Statistical Analysis Software) version 9.4 or higher, developed by SAS Institute, Inc., Cary, USA.

## Results

### Study population

The distribution of participants across the different study populations is presented in the participant flow diagram in Figure. During the study period, 6,233,729 of participants of all ages with at least one medical claim documented in the database were identified. These included 4,069,635 participants aged ≥ 20 years with at least one claim documented during the selection period. Overall, 1,402,247 had at least one diagnostic cough code (as listed in Table 1) documented during the selection period. Of these, 12,467 adult participants met the inclusion criteria; the remaining 1,389,780 participants were excluded principally because they did not have a documented cough diagnosis code that fulfilled the selection criteria or because they were younger than twenty years of age. A further 6,429 otherwise eligible participants were excluded, principally due to comorbid cancer. The remaining 6,038 participants constituted the chronic cough study population (Population ‘All’).

### Cough diagnosis at inclusion

**Fig. 2:**
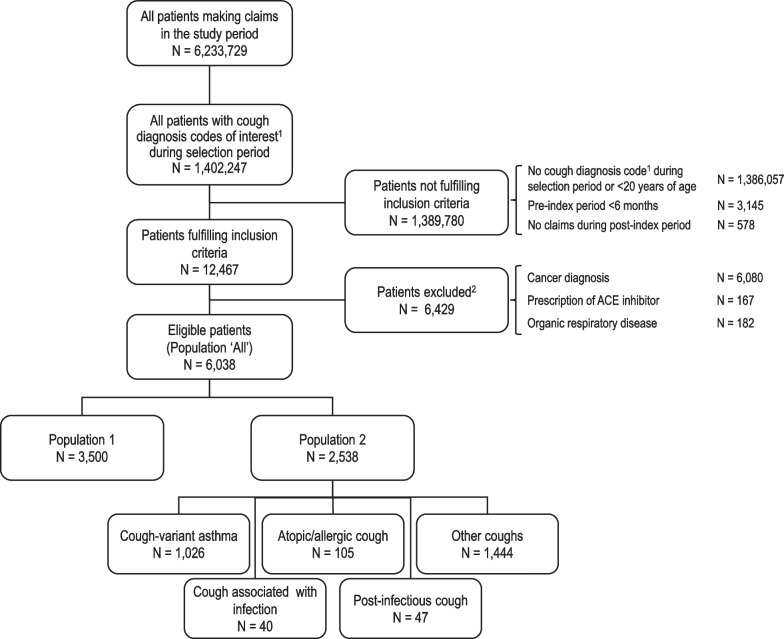
Study flow diagram. ^1^As defined in Table1. ^2^Medical conditions which are exclusion criteria for the study are listed in Additional file 1: Table 1. Study period: 1st January 2017 to 31st August 2020; selection period: 1st July 2017 and 31st August 2019. ACE: angiotensin-converting enzyme

These 6,038 participants in Population ‘All’ represent 0.15% of the 4,069,635 adult participants in the database making any medical claim during the selection period. Of the participants in Population ‘All’, 3,500 (58.0%) had an explicit diagnostic code for chronic cough documented in the database during the index month and constituted Population 1. The remaining 2,538 participants (42.0%) were assigned other ICD-10 diagnostic codes on the basis of the repeated presence of these codes in the database and constituted Population 2 (Fig. 2). Within Population 2, the most frequent cough subgroup was the ‘other coughs’ subgroup (*N* = 1,444), followed by cough-variant asthma (*N* = 1,026) and atopic/allergic cough (*N* = 105) (Fig. 2). The ‘cough associated with infections’ and ‘post-infectious cough’ subgroups accounted for less than sixty participants each, and less than one hundred participants overall.

Certain participants in each participant population and subgroups also had another documented diagnostic cough code during the index month (Table 2). In Population 1 this was most frequently ‘cough associated with infection’ (*N* = 145; 4.1%), cough-variant asthma (*N* = 126; 3.6%) and ‘other coughs’ (*N* = 125; 3.6%). In Population 2, these were no participants with a chronic cough diagnosis code since participants eligible for both Populations 1 and 2 were assigned de facto to Population 1. However, within the subgroups, multiple cough diagnoses were sometimes observed. For example, 60 participants in the ‘other coughs’ subgroup (4.2%) and 17 in the atopic/allergic cough subgroup (16.2%) also had a cough-variant asthma documented in the index month.

**Table 2:** Cough diagnoses during the index month

Population	Population ‘All’	Population 1	Population 2	Other coughs	Cough-variant asthma	Atopic/allergic cough
Number of participants	*N* = 6,038	*N* = 3,500	*N* = 2,538	*N* = 1,444	*N* = 1,026	*N* = 105
Chronic cough	3,500 (58.0%)	3,500 (100%)	0	0	0	0
Cough associated with infection	185 (3.1%)	145 (4.1%)	40 (1.6%)	9 (0.6%)	8 (0.8%)	1 (1.0%)
Atopic cough / allergic cough	131 (2.2%)	31 (0.9%)	100 (3.9%)	5 (0.3%)	14 (1.4%)	100 (95.2%)*
Cough-variant asthma	1,131 (18.7%)	126 (3.6%)	1,005 (39.6%)	60 (4.2%)	1,003 (97.8%)*	17 (16.2%)
Postinfectious cough	58 (1.0%)	11 (0.3%)	47 (1.9%)	4 (0.3%)	4 (0.4%)	0
Other coughs	1,532 (25.4%)	125 (3.6%)	1,407 (55.4%)	1,407 (97.4%)*	47 (4.6%)	3 (2.9%)

^*^These values are < 100% due to differences in how the subgroups and the cough diagnoses during the index month are assigned. In the subgroups, the definition was based on the presence of the relevant diagnostic cough code between the first diagnoses of cough (potentially six months prior to the index month) and the index month itself, while the cough diagnosis during the index month requires the presence of the relevant diagnostic cough code specifically during the index month

In the different subgroups of Population 2, it could also arise that the cough diagnosis associated with the subgroup was not documented during the index month, but only during the 3-month evaluation period. For example, only 1,003 of the 1,026 participants in the cough-variant asthma subgroup had a diagnosis of cough-variant asthma documented during the index month (Table 2). However, these cases were rare and accounted for < 5% of participants in each subgroup.

In Population 1, all 3,500 participants had a documented chronic cough diagnosis code during the index month, since this was a requirement of the eligibility criteria.

### Participant characteristics at inclusion

Participant characteristics at inclusion in the populations of interest are summarised for all chronic cough populations and subgroups in Table 3. The mean age of the participants in Population ‘All’ was 43.7 years and women were over-represented compared to men (61.8% overall). The majority of the participants (69.9% overall) consulted in a clinic with less than 20 beds, rather than in a hospital. A minority of participants (21.4%) were referred to large hospitals having more than 200 beds.

**Table 3:** Participant characteristics at the index month

Population		Population ‘All’	Population 1	Population 2	Other coughs	Cough-variant asthma	Atopic/allergic cough
Number of patients	*N* = 6,038	*N* = 3,500	*N* = 2,538	*N* = 1,444	*N* = 1,026	*N* = 105	
*Age at index date*							
	Mean ± SD	43.7 ± 12.2	42.3 ± 12.2	45.6 ± 11.9	45.3 ± 11.9	45.8 ± 11.8	46.2 ± 11.8
	≥ 60 years	564 (9.3%)	283 (8.1%)	281 (11.1%)	153 (10.6%)	124 (12.1%)	10 (9.5%)
*Gender*							
	Men	2,308 (38.2%)	1,402 (40.1%)	906 (35.7%)	503 (34.8%)	369 (36.0%)	42 (40.0%)
	Women	3,730 (61.8%)	2,098 (59.9%)	1,632 (64.3%)	941 (65.2%)	657 (64.0%)	63 (60.0%)
*Medical facility size*	*N* = 6,000	*N* = 3,483	*N* = 2,517	*N* = 1,444	*N* = 1,017	*N* = 105	
	1–19 beds	4,196 (69.9%)	2,197 (63.1%)	1,999 (79.4%)	1,202 (83.9%)	783 (77.0%)	65 (61.9%)
	20–199 beds	521 (8.7%)	373 (10.7%)	148 (5.9%)	46 (3.2%)	85 (8.4%)	10 (9.5%)
	≥ 200 beds	1,283 (21.4%)	913 (26.2%)	370 (14.7%)	184 (12.8%)	149 (14.7%)	30 (28.6%)
*Cough-related diseases*							
	ARNI	3,132 (51.9%)	1,688 (48.2%)	1,444 (56.9%)	776 (53,7%)	619 (60.3%)	71 (67,6%)
	Asthma	2,517 (41.7%)	1,620 (46.3%)	897 (35.3%)	506 (35,0%)	340 (33.1%)	54 (51,4%)
	GERD	829 (13.7%)	448 (12.8%)	381 (15.0%)	185 (12,8%)	179 (17.5%)	20 (19,0%)
	Paranasal sinusitis	724 (12.0%)	455 (13.0%)	269 (10.6%)	170 (11,8%)	89 (8.7%)	8 (7,6%)
	CAID	482 (8.0%)	312 (8.9%)	170 (6.7%)	91 (6,3%)	68 (6.6%)	10 (9,5%)
	Chronic rhinitis	155 (2.6%)	79 (2.3%)	76 (3.0%)	59 (4,1%)	19 (1.9%)	0
	Postnasal drip	50 (0.8%)	22 (0.6%)	28 (1.1%)	3 (0,2%)	25 (2.4%)	1 (1,0%)
	Vocal cord dysfunction	74 (1.2%)	46 (1.3%)	28 (1.1%)	15 (1,0%)	13 (1.3%)	0
	Emphysema	48 (0.8%)	39 (1.1%)	9 (0.4%)	5 (0,3%)	3 (0.3%)	1 (1,0%)
	Nasal polyps	6 (0.1%)	4 (0.1%)	2 (0.1%)	2 (0,1%)	0	0

*ARNI* Allergic rhinitis/nasal inflammation, *CAID* Chronic airway inflammatory disease, *GERD* Gastro-esophageal reflux disease, *IQR* interquartile range, *SD* Standard deviation

Regarding the cough-related diseases of interest documented during the index month, the most frequently reported in Population ‘All’ were allergic rhinitis/nasal inflammation (51.9%), asthma (41.7%), GERD (13.7%), paranasal sinusitis (12.0%) and chronic airway inflammatory disease (8.0%). No other cough-related disease was reported in > 3% of participants.

### Stability of cough diagnosis over time

Chronic cough can persist for many years despite appropriate treatments for underlying diseases. In order to understand if cough-related diseases and drug prescription patterns change over time, we described cough diagnosis, cough-related disease and medications of participants longitudinally which can be compared with baseline conditions. In Months 10 to 12, the number of eligible participants with a documented cough diagnosis had declined (Table 4). In Population 1, only 470 participants had a documented diagnostic code for chronic cough in Months 10–12. This represents 36.6% of the 1,284 participants with a documented cough diagnosis or diagnosis of a cough-related disease during this period. As was the case in the index month, less than five percent of participants in Population 1 had a diagnostic code for another type of cough.

**Table 4:** Cough diagnoses documented at Months 10 to 12

Population/subgroup	Population ‘All’	Population 1	Population 2	Other coughs	Cough-variant asthma	Atopic/allergic cough
Participants enrolled	*N* = 6,038	*N* = 3,500	*N* = 2,538	*N* = 1,444	*N* = 1,026	*N* = 105
Participants evaluable at M10-12^1^	*n* = 2,804	*n* = 1,284	*n* = 1,520	*n* = 856	*n* = 615	*n* = 63
Cough diagnosis in M10-M12						
Chronic cough	472 (16.8%)	470 (36.6%)	2 (0.1%)	2 (0.2%)	0	0
Other coughs	527 (18.8%)	46 (3.6%)	481 (31.6%)	474 (55.4%)	16 (2.6%)	1 (1.6%)
Cough-variant asthma	477 (17.0%)	36 (2.8%)	441 (29.0%)	32 (3.7%)	427 (69.4%)	7 (11.1%)
Atopic/allergic cough	49 (1.8%)	7 (0.6%)	42 (2.8%)	1 (0.1%)	5 (0.8%)	41 (65.1%)
Cough associated with infection	19 (0.7%)	9 (0.7%)	10 (0.7%)	4 (0.5%)	2 (0.3%)	1 (1.6%)
Post-infectious cough	19 (0.7%)	6 (0.5%)	13 (0.9%)	1 (0.1%)	2 (0.3%)	0

^1^Defined as those participants with a cough diagnosis code or a diagnostic code for a cough-related disease during the 3-month period from Month 10 to Month 12 inclusive

Proportions are calculated with the respect to the number of participants evaluable in Months 10 to 12 (M10-12)

In Population 2, only two participants acquired a diagnostic code for chronic cough. In the ‘other coughs’ subgroup of Population 2, the proportion of participants documented at Months 10–12 who had a diagnosis of ‘other coughs’ during this period was 55.4% (Table 4). The corresponding figures for the other subgroups were 69.4% for cough-variant asthma and 65.1% for atopic/allergic cough. In all subgroups, the number (and proportion) of participants with a different type of cough diagnosis during Months 10–12 was low (Table 4).

### Cough-related diseases

In order to investigate what cough-related diseases were present prior to the first documentation of chronic cough, and to observe how the frequency of these underlying cough-related diseases changed over time, we determined the proportion of participants with cough-related diseases during the six-month period leading up to and including the index month (referred to as the pre-index period) and during Months 10 to 12 of the post-index period (Table 5). In Population ‘All’, the proportion of participants with allergic rhinitis/nasal inflammation was 63.4% during the pre-index period and 70.4% during Months 10 to 12, and the proportion of participants with asthma was 48.4% and 42.0% respectively (see Table 5 in bold). These changes were essentially driven by Population 1. In the atopic/allergic cough subgroup, there was a small increase in the proportion of participants with GERD at Months 10 to 12 compared to the pre-index period and a reduction in that of participants with asthma, although it should be noted that participant numbers in this subgroup were low.

**Table 5:** Cough-related diseases in the preindex period and in Months 10 to 12 of the post-index period

Population/ subgroup	Population ‘All’		Population 1		Population 2		Other coughs		Cough-variant asthma		Atopic/allergic cough	
Period	Pre-index	M10-12	Pre-index	M10-12	Pre-index	M10-12	Pre-index	M10-12	Pre-index	M10-12	Pre-index	M10-12
Number of participants^1^	*N* = 6,038	*N* = 2,804	*N* = 3,500	*N* = 1,284	*N* = 2,538	*N* = 1,520	*N* = 1,444	*N* = 856	*N* = 1,026	*N* = 615	*N* = 105	*N* = 63
ARNI	**3,826 (63.4%)**	**1,975 (70.4%)**	**2,093 (59.8%)**	**899 (70.0%)**	1,733 (68.3%)	1,076 (70.8%)	950 (65.8%)	600 (70.1%)	737 (71.8%)	448 (72.8%)	77 (73.3%)	44 (69.8%)
Asthma	**2,920 (48.4%)**	**1,179 (42.0%)**	**1,778 (50.8%)**	**533 (41.5%)**	1,142 (45.0%)	646 (42.5%)	640 (44.3%)	358 (41.8%)	453 (44.2%)	259 (42.1%)	**64 (61.0%)**	**31 (49.2%)**
GERD	1,029 (17.0%)	574 (20.5%)	552 (15.8%)	256 (19.9%)	477 (18.8%)	318 (20.9%)	246 (17.0%)	153 (17.9%)	211 (20.6%)	149 (24.2%)	**27 (25.7%)**	**20 (31.7%)**
Paranasal sinusitis	1,037 (17.2%)	439 (15.7%)	621 (17.7%)	210 (16.4%)	416 (16.4%)	229 (15.1%)	260 (18.0%)	147 (17.2%)	149 (14.5%)	75 (12.2%)	18 (17.1%)	8 (12.7%)
CAID	586 (9.7%)	217 (7.7%)	366 (10.5%)	101 (7.9%)	220 (8.7%)	116 (7.6%)	123 (8.5%)	66 (7.7%)	88 (8.6%)	39 (6.3%)	14 (13.3%)	6 (9.5%)
Chronic rhinitis	206 (3.4%)	106 (3.8%)	106 (3.0%)	46 (3.6%)	100 (3.9%)	60 (3.9%)	72 (5.0%)	43 (5.0%)	31 (3.0%)	18 (2.9%)	0	0
Postnasal drip	127 (2.1%)	42 (1.5%)	78 (2.2%)	26 (2.0%)	49 (1.9%)	16 (1.1%)	27 (1.9%)	9 (1.1%)	21 (2.0%)	8 (1.3%)	1 (1.0%)	0
Vocal cord dysfunction	71 (1.2%)	21 (0.7%)	33 (0.9%)	8 (0.6%)	38 (1.5%)	13 (0.9%)	8 (0.6%)	1 (0.1%)	30 (2.9%)	12 (2.0%)	1 (1.0%)	0
Emphysema	62 (1.0%)	13 (0.5%)	43 (1.2%)	8 (0.6%)	19 (0.7%)	5 (0.3%)	12 (0.8%)	2 (0.2%)	5 (0.5%)	2 (0.3%)	2 (1.9%)	1 (1.6%)
Nasal polyps	10 (0.2%)	5 (0.2%)	6 (0.2%)	3 (0.2%)	4 (0.2%)	2 (0.1%)	2 (0.1%)	1 (0.1%)	2 (0.2%)	1 (0.2%)	0	0

^1^Defined as those participants with a cough diagnosis code or a diagnostic code for a cough-related disease during the 6-month pre-index period including the index month (pre-index period), or during the 3-month period from Month 10 to Month 12 inclusive following the index month (M10-12)

Percentages are calculated with respect to the number of participants indicated at the top of the column

Data in **bold** indicate a difference of > 5% in the proportion of participants with the cough-related disease between the pre-index period and M10-12

*ARNI* Allergic rhinitis/nasal inflammation, *CAID* Chronic airway inflammatory disease, *GERD* Gastro-esophageal reflux disease

### Medication use related to chronic cough

During the index month, 4,860 participants (80.5% of the number of patients in the cohort) in Population ‘All’ were prescribed at least one treatment of interest (Table 6). Prescription of these medications occurred in a greater proportion of Population 1 (89.0%) than in Population 2 (68.8%). Central antitussives alone were prescribed in 194 participants (4.0%), the majority of whom were in Population 1 (169 participants; 87.1% of participants prescribed antitussives alone). In Population ‘All’, expectorants were prescribed to 2,331 participants (48.0%), more frequently in Population 1 (53.1%) than in Population 2 (38.8%) (Table 6).

**Table 6:** Treatments prescribed during the index month

	Population ‘All’	Population 1	Population 2	Other coughs	Cough-variant asthma	Atopic/allergic cough
	*N* = 6,038	*N* = 3,500	*N* = 2,538	*N* = 1,444	*N* = 1,026	*N* = 105
Any medication of interest	4,860 (80.5%)	3,115 (89.0%)	1,745 (68.8%)	997 (69.0%)	722 (70.4%)	76 (72.4%)
Central antitussives only	194 (4.0%)	169 (5.4%)	25 (1.4%)	21 (2.1%)	4 (0.6%)	0 (0.0%)
Antiallergic agents (all)	2,588 (53.3%)	1,628 (52.3%)	960 (55.0%)	503 (50.5%)	429 (59.4%)	52 (68.4%)
Without central antitussives	2,493 (51.3%)	1,544 (49.6%)	949 (54.4%)	496 (49.7%)	427 (59.1%)	51 (67.1%)
In combination with central antitussives	95 (2.0%)	84 (2.7%)	11 (0.6%)	7 (0.7%)	2 (0.3%)	1 (1.3%)
Expectorant (all)	2,331 (48.0%)	1,654 (53.1%)	677 (38.8%)	402 (40.3%)	281 (38.9%)	24 (31.6%)
Without central antitussives	1,990 (40.9%)	1,392 (44.7%)	598 (34.3%)	348 (34.9%)	256 (35.5%)	21 (27.6%)
In combination with central antitussives	341 (7.0%)	262 (8.4%)	79 (4.5%)	54 (5.4%)	25 (3.5%)	3 (3.9%)
Antimicrobials^a^ (all)	1,672 (34.4%)	1,245 (40.0%)	427 (24.5%)	271 (27.2%)	161 (22.3%)	12 (15.8%)
Without central antitussives	1,224 (25.2%)	886 (28.4%)	338 (19.4%)	205 (20.6%)	138 (19.1%)	10 (13.2%)
In combination with central antitussives	448 (9.2%)	359 (11.5%)	89 (5.1%)	66 (6.6%)	23 (3.2%)	2 (2.6%)
ICS and LABA combination (all)	1,404 (28.9%)	901 (28.9%)	503 (28.8%)	198 (19.9%)	309 (42.8%)	25 (32.9%)
Without central antitussives	1,049 (21.6%)	622 (20.0%)	427 (24.5%)	158 (15.8%)	265 (36.7%)	23 (30.3%)
In combination with central antitussives	355 (7.3%)	279 (9.0%)	76 (4.4%)	40 (4.0%)	44 (6.1%)	2 (2.6%)
Herbal medicine (all)	1,113 (22.9%)	781 (25.1%)	332 (19.0%)	264 (26.5%)	67 (9.3%)	8 (10.5%)
Without central antitussives	1,047 (21.5%)	723 (23.2%)	324 (18.6%)	258 (25.9%)	66 (9.1%)	6 (7.9%)
In combination with central antitussives	66 (1.4%)	58 (1.9%)	8 (0.5%)	6 (0.6%)	1 (0.1%)	2 (2.6%)
Corticosteroids (all)	1,080 (22.2%)	636 (20.4%)	444 (25.4%)	259 (26.0%)	176 (24.4%)	15 (19.7%)
Without central antitussives	716 (14.7%)	366 (11.7%)	350 (20.1%)	197 (19.8%)	150 (20.8%)	11 (14.5%)
In combination with central antitussives	364 (7.5%)	270 (8.7%)	94 (5.4%)	62 (6.2%)	26 (3.6%)	4 (5.3%)
Bronchodilators^b^ (all)	914 (18.8%)	694 (22.3%)	220 (12.6%)	124 (12.4%)	99 (13.7%)	8 (10.5%)
Without central antitussives	287 (5.9%)	170 (5.5%)	117 (6.7%%))	62 (6.2%)	60 (8.3%)	2 (2.6%)
In combination with central antitussives	627 (12.9%)	524 (16.8%)	103 (5.9%)	62 (6.2%)	39 (5.4%)	6 (7.9%)
Drugs for peptic ulcer treatment (all)	718 (14.8%)	436 (14.0%)	282 (16.2%)	145 (14.5%)	127 (17.6%)	18 (23.7%)
Without central antitussives	691 (14.2%)	412 (13.2%)	279 (16.0%)	143 (14.3%)	126 (17.5%)	18 (23.7%)
In combination with central antitussives	27 (0.6%)	24 (0.8%)	3 (0.2%)	2 (0.2%)	1 (0.1%)	0 (0.0%)
Drugs for improvement of GI motility function (all)	210 (4.3%)	107 (3.4%)	103 (5.9%)	55 (5.5%)	43 (6.0%)	8 (10.5%)
Without central antitussives	205 (4.2%)	102 (3.3%)	103 (5.9%)	55 (5.5%)	43 (6.0%)	8 (10.5%)
In combination with central antitussives	5 (0.1%)	5 (0.2%)	0 (0.0%)	0 (0.0%)	0 (0.0%)	0 (0.0%)

*ICS* Inhaled corticosteroids, *LABA* Long-acting beta-adrenergic receptor antagonists, *GI* Gastrointestinal

All percentages are expressed using the number of participants taking any medication of interest as the denominator

^a^Respiratory quinolones and 14- and 15-member ring macrolides only

^b^Excluding participants using long-acting beta-adrenergic receptor antagonists in combination with inhaled corticosteroids

Of the medications prescribed to treat common causes of chronic cough in Population ‘All’ during the index months, the most frequently documented classes were antiallergic agents (*N* = 2,588; 53.3%), antimicrobials (*N* = 1,672; 34.4%) and combinations of ICS and LABA (*N* = 1,404; 28.9%). Medication classes used more frequently (between-population difference ≥ 5%) in Population 1 than in Population 2 were bronchodilators, antimicrobials and herbal medicines, whereas corticosteroids were used more frequently in Population 2 (Table 6). Within Population 2, antiallergic agents (*N* = 429; 50,4%) and ICS + LABA (*N* = 309; 42.8%) were frequently used in patients in the cough-variant asthma subgroup. These two classes were also frequently used in the atopic/allergic cough subgroup (antiallergic agents: *N* = 52; 68.4%) and (ICS + LABA: *N* = 25; 32.9%). In terms of the use of combination therapy with central antitussives, this was more frequent in Population 1 than in Population 2 for all medication classes. Within Population 2, prescription in combination with a central antitussive was more frequent in the atopic/allergic cough group for the bronchodilator and corticosteroid medication classes (Table 6). The bronchodilator class was the only class that was used in combination with a central antitussive (*N* = 627; 12.9%) more often than not (*N* = 287; 5.9%) in Population ‘All’, the majority of whom were in Population 1 (*N* = 524; 16.8% with central antitussives, *N* = 170; 5.5% without central antitussives) (Table 6).

**Fig. 3:**
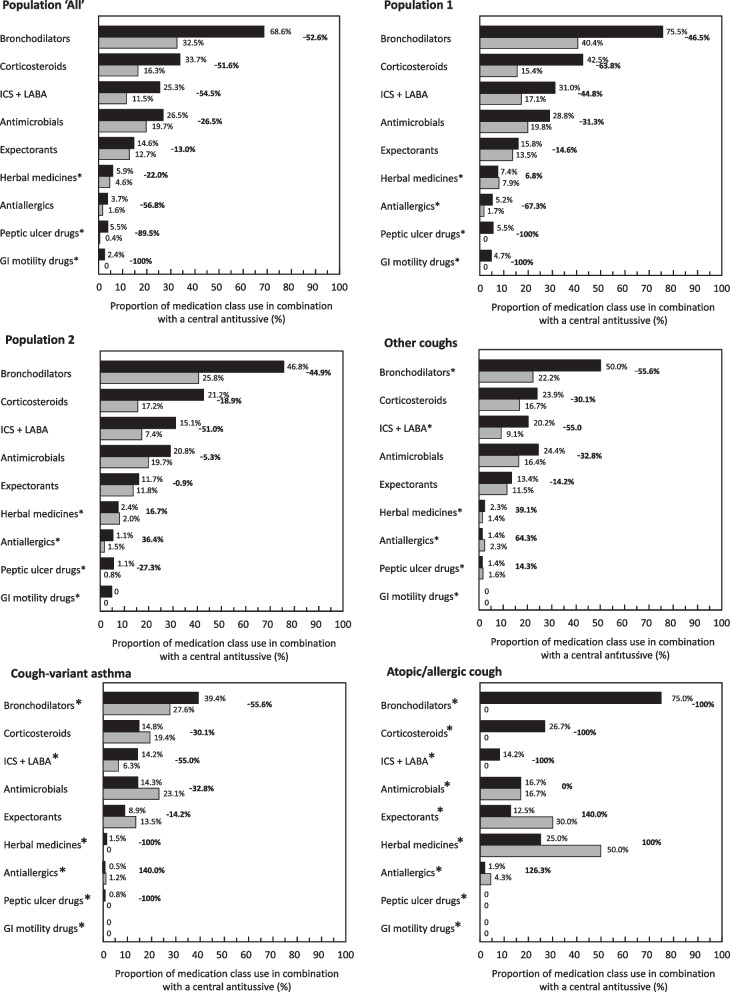
Use of combination therapy with a central antitussive at index month and at Month 12. ICS: inhaled corticosteroids; LABA: long-acting beta-adrenergic receptor antagonists. Antimicrobials refer to respiratory quinolones and 14- and 15-member ring macrolides only. Black box: Index month; White box: Month 12. The total number of participants with a prescription claim for a medication of interest at Month 12 was 1,317 in Population ‘All’, 628 in Population 1, 689 in Population 2, 394 in the ‘other coughs’ subgroup, 281 in the cough-variant asthma subgroup and 32 in the atopic/allergic cough group. The proportion of medication use in combination is calculated as the number of participants using a given medication class in combination with a central antitussive divided by the total number of users of the given class. The percentages to the right of each pair of bars represent the change (%) in the proportion of medication use in combination between the index month and Month 12. The asterisk indicates the medication classes which were used in combination with corticosteroids in less than 10 patients at either time point

**Fig. 4:**
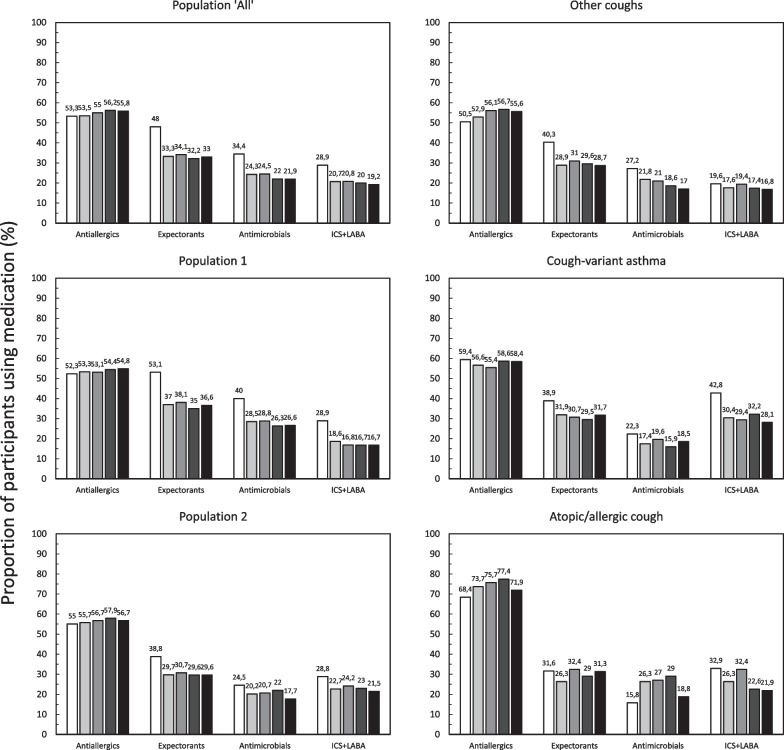
Changes in medication prescription over the post-index period. ICS: inhaled corticosteroids; LABA: long-acting beta-adrenergic receptor antagonists. Antimicrobials refer to respiratory quinolones and 14- and 15-member ring macrolides only. White box: Index month (*N* = 4,860); Black box Month 3 (*N* = 1,693); Black box Month 6 (*N* = 1,491); Black box Month 9 (*N* = 1,392); Black box Month 12 (*N* = 1,317). All percentages are expressed using the number of participants taking any medication of interest as the denominator (either alone or in combination with central antitussives). Full data are provided in Additional file 1: Table 3

In order to understand how central antitussives were used during the chronic phase of cough, the proportion of patients receiving a central antitussive in addition to a medication used to treat cough-related diseases was calculated in each medication class, which was then compared between the index month (Fig. 3, black bars) and Month 12 (Fig. 3, gray bars). For Population ‘All’, 68.6% (*N* = 627) of the 914 patients prescribed bronchodilators were also prescribed a central antitussive (Table 6 and Fig. 3). For the other medication classes, patients prescribed corticosteroids (33.7%), antimicrobials (26.5%), ICS + LABA (25.3%) and expectorants (14.6%) were also prescribed a central antitussive (Table 6 and Fig. 3, black bars). In Population ‘All’, the proportion of participants prescribed medications used to treat cough-related diseases in combination with a central antitussive fell between the index month and Month 12 for all medication classes, in most cases by a factor of at least two (Fig. 3). This decrease was observed in both Population 1 and Population 2 (Fig. 3). A similar pattern was observed for all the three subgroups of Population 2, although these findings should be interpreted with caution given the very low number of users for certain medication classes. (Fig. 4)

Medication use over the post-index period was evaluated, and the proportion of users expressed as a percentage of those participants with any prescription of a medication of interest documented in the database during the month in question. In the months following the index month, the proportion of participants prescribed a central antitussive alone fell sharply (Table 7). In Population `All`, the proportion of medication users prescribed only central antitussives fell from 4.0% during the index month to 1.1% in Month 12. In Population 1, which accounted for most of these prescriptions, the proportion of medication users prescribed central antitussives fell from 5.4% during the index month to 1.1% in Month 12.

**Table 7:** Prescription of central antitussives alone during the post-index period

Population	Population ‘All’	Population 1	Population 2	Other coughs	Cough-variant asthma	Atopic/allergic cough
Number of participants	*N* = 6,038	*N* = 3,500	*N* = 2,538	*N* = 1,444	*N* = 1,026	*N* = 105
Index month	*n* = 4,860 194 (4.0%)	*n* = 3,115 169 (5.4%)	*n* = 1,745 25 (1.4%)	*n* = 997 21 (2.1%)	*n* = 722 4 (0.6%)	*N* = 76 0
M3	*n* = 1 693 19 (1.1%)	*n* = 827 10 (1.2%)	*n* = 866 9 (1.0%)	*n* = 499 7 (1.4%)	*n* = 339 2 (0.6%)	*n* = 38 0
M6	*n* = 1 491 22 (1.5%)	*n* = 695 16 (2.3%)	*n* = 796 6 (0.8%)	*n* = 458 4 (0.9%)	*n* = 316 1 (0.3%)	*n* = 37 1 (2.7%)
M9	*n* = 1 392 21 (1.5%)	*n* = 666 13 (2.0%)	*n* = 726 8 (1.1%)	*n* = 409 5 (1.2%)	*n* = 295 3 (1.0%)	*n* = 31 0
M12	*n* = 1 317 14 (1.1%)	*n* = 628 7 (1.1%)	*n* = 689 7 (1.0%)	*n* = 394 1 (0.3%)	*n* = 281 6 (2.1%)	*n* = 32 0

*N* represents the total number of participants enrolled in each group; n represents the number of participants with at least one prescription claim for a medication of interest in each month. The latter number was used as the denominator for the calculation of the percentages

The proportion of medication users prescribed expectorants in Population ‘All’ decreased from 48.0% during the index month to 32.9% in Month 12. For the other medication classes, their prescription remained relatively stable throughout the post-index period (Fig. 4), although there was a decrease in the proportion prescribed antimicrobials and ICS-LABA following the index month to a lower plateau. This pattern was similar across study populations and subgroups, although changes were more pronounced in Population 1 compared to Population 2.

Complete information on medication use in the different participant subgroups throughout the post-index period is provided in Additional file 1: Table 3.

## Discussion

This study describes the characteristics of individuals with chronic cough, their underlying cough-related diseases and their treatments using a Japanese claims database. We devised an original algorithm to define the population of patients managed for chronic cough. This enabled us to identify a total of 6,038 such patients over the 26-month selection period.

Chronic cough is not explicitly defined in the current (2021) international classification of diseases of the World Health Organization and there is thus no single specific ICD-10 code which can be used to capture the entire chronic cough population, nor any established algorithm to identify patients with chronic cough in insurance claims databases. For these reasons, a major challenge of this study was to create original algorithms to identify these patients within the constraints of the coding conventions used in the Japanese healthcare system. The algorithm used took into account a combination of the ICD-10 codes and the more specific standard disease names in Japanese vernacular documented for each physician visit, and the frequency of visits with a cough diagnosis code. As well as including patients with chronic cough identified by the standard disease name in Japanese, the algorithm also selected patients with other cough diagnoses that were documented at least three times over a 6-month period. Due to the particularity of the Japanese insurance claims system, whereby consultations are documented on a monthly (rather than daily) basis, it was not possible to document the exact date of consultation within each month and, for this reason, the algorithm used the more stringent criterion of multiple consultations over at least 3 months in order to ensure that the eight-week criterion was met. While antitussive use for ≤ 8 weeks could be considered a straightforward marker for a chronic cough population, we chose not take into account this criterion in the selection algorithm, because of the risk of false positives due to potential use of certain medications as central antitussives or analgesics.

It should also be noted that many patients with chronic cough do not regularly consult a physician for their cough. In an internet survey of patients with cough performed in Japan in 2011, over 60% of interviewees were not being treated for their cough and 44% did not envisage consulting a physician about it [12]. For this reason, our selection algorithm may only identify the more severe patients who were actively seeking treatment for their cough symptoms and thus carry a high disease burden.

The 6,038 patients with chronic cough identified represent 0.15% of all the patients in the Medi-Scope® database who made an insurance claim during the selection period. However, this proportion should not be considered as a true prevalence rate since patients in the database not making claims or not consulting a physician are not visible and the true number of individuals with chronic cough is thus not known. A recent general population web-based survey in Japan reported a point prevalence of self-reported chronic cough in adults of 2.9% and a 12-month prevalence of 4.3% [15], consistent with a previous survey reporting a point prevalence of 2.4% [12]. Recent data from Europe and North America suggest a similar prevalence of about 4 to 5% [16–18], although higher rates have been reported in earlier studies [24]. Compared to these prevalence rates, the proportion of patients in our sample fulfilling the criteria for chronic cough is low, which may reflect the fact that our sample is restricted to those patients actively seeking treatment for their coughs from a physician and does not include patients who treat themselves or seek help from other healthcare professionals such as pharmacists. In addition, it is possible that patients with cough due to underlying disease are documented in the Medi-Scope® database by the code of the underlying disease rather than a cough code, and these patients will not be captured by our algorithm. Identifying patients who have a claim related to an underlying disease together with a prescription for a cough medication lasting more than 8 weeks could provide us another way to identify patients with chronic cough. However, this definition would carry a risk of false positives due to noise from the prescription of antitussives for other diseases. Since we have no established algorithms to eliminate that noise so that we prioritised fidelity rather than uncertainty. Although not all patients will be captured, we chose to use the algorithm based on diagnostic codes which we believe should have high specificity for chronic cough.

Study participants with chronic cough were more frequently women than men, consistent with previous reports from other countries [11, 14, 16, 17, 25], but surprisingly not with two internet surveys from Japan, which both reported a higher prevalence in men [12, 15]. The mean age of our population (44 years in Population ‘All’) is rather low compared to that reported in previous studies (> 50 years) [11, 15, 17, 25], but this difference is probably to be explained by the age structure of the Medi-Scope® database, which insures employees and their families, so the number of retired people in the database aged > 65 years is low. With regard to underlying cough-related diseases, their relative proportion was similar to the results of patients’ self-reported internet survey in the Japanese general population [15], although absolute proportions were higher in the present study. This difference may be explained by the fact that our population is actively seeking care for their chronic cough and may thus have more severe disease. The proportion of patients presenting these cough-related diseases remained relatively stable over the course of the present study.

Over the post-index follow-up period, diagnostic cough codes were frequently no longer documented during the post-index period. For example, in Population 1, of the 3,500 participants with a diagnostic code for chronic cough at inclusion, only 1,284 particpants retained a diagnosis code or a cough related disease code at Months 10–12 (Table 4). Regarding the remaining 2,216 patients who left the cohort, several reasons for their withdrawal could be suggested: 1) The cough could have resolved during the post-index period, either spontaneously or following treatment, 2) Some patients may have left the database during the year following the index date. The database provider has estimated that around 14% of patients in the database at any given time leave the database within a one-year time period, 3) Certain participants may no longer consult for their cough and manage it with non-prescription medication, and 4) Some of these patients may been diagnosed with an underlying disease that was not retained in our list of cough-related disorders. Same explanation can apply to other cough diagnosis groups such as cough-variant asthma group as well (Table 4).

Of the 1,284 patients in Population 1 who do retain a diagnostic cough code or a cough-related disease code at Months 10–12, 470 (36.6%) retain their original code for chronic cough (Table 4). The rest of patients, 104 (8.1%) (a sum of patients with other coughs (46), cough-variant asthma (36), atopic/allergic cough (7), cough associated with infection (9) and post-infectious cough (6) in Table 4) was assigned another diagnostic cough code. 710 (55.3%) who do not have any diagnostic cough code move to a cough-related disease code only (Table 5). However, cough-variant asthma group, 427 (69.4%) retain their original code, 25 (4.1%) was assigned another diagnostic cough code and 163 (26.5%) move to a cough-related disease code only (Tables 4 and 5). These suggested that cough-variant asthma with an initial diagnosis were infrequently reassigned a cough-related disease code but patients in Population 1 with an initial diagnosis code were frequently subsequently reassigned a cough-related disease code (Table5). It may suggest that the underlying cause of the chronic cough has been identified in Population1, perhaps through appropriate diagnostic work-up. Further, some clinicians may have waited to document a cough-related disease code until they were confident of the underlying cause of cough where others may have used diagnosis codes to document the presence of a cough symptom. Regarding cough-variant asthma group, cough-variant asthma itself could be the underlying cause of the chronic cough. In addition, it is also possible that certain participants have been re-diagnosed as a symptom of another disease which is not in our algorithm.

The proportion of participants whose chronic cough persisted for a year (36.6% in Population 1) is lower than that reported in a recent study from the USA, in which 41% of patients with chronic cough retained their cough diagnosis 12 months later [3]. However, the populations of the two studies were different, with almost half of the US population having GERD, compared to 17% in the present study. In addition, in the US study, the specialty of the physicians consulted at baseline influenced the reported persistence of chronic cough. In particular, an allergist visit was associated with lower persistence and a pulmonologist visit associated with higher persistence [3]. The relationship between physician speciality and persistence of chronic cough would be of interest to evaluate in the present study population.

With regard to treatment, the proportion of participants prescribed a central antitussive alone during the index month was very low (4.0% overall). This is consistent with the Japanese respiratory society guidelines for the management of cough [19], which discourage use of these agents due to adverse effects such as constipation and drowsiness. Expectorants represent the other class of specific antitussive medication, and these treatments were widely prescribed, to 48% of participants during the index month and to around 33% each month during the post-index period. This indicates that sputum control is an important element of the standard care of chronic cough in Japan, consistent with practice guidelines.

Japanese cough management guidelines recommend treating the underlying disease rather than prescribing central antitussives [19]. These recommendations appear to have been followed, since the proportion of participants prescribed these medications without central antitussives was 51.3% for antiallergic agents, 42.2% for corticosteroids or bronchodilators (including 14.7% of corticosteroids, 21.6% of ICS + LABA and 7.5% of bronchodilators) and 18.4% for peptic ulcer treatments (including 4.2% of drugs for improvement of gastrointestinal mobility); these numbers are consistent with the 51.9% of participants with allergic rhinitis/nasal inflammation, 41.7% with asthma and 13.7% with GERD (Tables 3 and 6 in Population `All`). Antiallergic agents and ICS + LABA were frequently used in patients in the cough-variant asthma subgroup as well as in the atopic/allergic cough subgroup, suggesting that the same combination of treatments was frequently used for two diseases with distinct disease mechanisms. In addition, these prescriptions were in general not renewed over the post-index follow-period, with very few participants still being prescribed antitussives six or twelve months later. Again, this is consistent with practice guidelines which discourage the long-term use of these agents. The principal difference in medication use between Populations 1 and 2 is the higher use of specific cough medications (central antitussives and expectorants) in Population 1.

Medications to treat underlying diseases were sometimes prescribed together with central antitussives at the index month; however, this only concerned > 20% of participants in the case of bronchodilators (including ICS + LABA), corticosteroids and antimicrobials. The proportion of participants co-prescribed a central antitussive fell over the course of the study.

The limitations are principally related to the information in the database. In particular, individual patient records with the results of examinations or laboratory tests are not available, and this precludes validating the diagnosis in the database or determining the aetiology of the cough. Moreover, information is unavailable on the temporal sequence of disease occurrence in individual patients in the database, and for this reason, it is not possible to distinguish between the underlying diseases that caused the cough and those that are independent comorbidities. Another limitation of the database is that patients who do not consult a physician and make a claim are not captured, nor is use of non-prescription medications. The database only covers employees and their families, so retired people over 65 years of age are under-represented. Finally, the total number of insurees in the database varies over time as individuals move into and out of the health insurance scheme. The only information available on the size of the source population is the number of beneficiaries making a claim in over a given period. For this reason, an accurate estimate of cough prevalence is not possible. In addition, patients who do not make a claim cannot be distinguished from those who leave the database for whatever reason (for example if they die or change employment), and it cannot be excluded that certain participants who were identified during the index month may leave the database during the post-index period because they changed their insurance plan. The database provider has estimated that around 14% of patients with an insurance plan documented in the database are transferring outside within a one-year time period.

A problem faced with identifying patients with chronic cough in this study was the difficulty in capturing patients with a cough lasting at least eight weeks. This arises because, in Japanese reimbursement databases, information on diagnosis is consolidated monthly in the database and we thus do not know the exact dates on which visits are made. For this reason, we used a more stringent criterion in order to ensure that the eight-week criterion was fulfilled (two visits in two different months during a three-month period, followed by a third visit in the following three months). Our criterion is likely to be highly specific for the formal eight-week criterion, but perhaps not fully sensitive. For this reason, it probably does not identify all patients with chronic cough who fulfil the formal eight-week criterion, although this cannot be demonstrated.

In conclusion, in this comprehensive claim database study, we were able to identify a population of patients seeking treatment for chronic cough and describe their characteristics, underlying cough-related diseases and drug utilisation patterns. The present study noted a generally good adherence to the standard of care for chronic cough patients recommended in JRS guideline. However, despite adequate treatments, chronic cough may persist for a year, suggesting the presence of unmet medical needs. Studies to identify the characteristics of patients with chronic cough and their care trajectory, as well as medical education about chronic cough would be further required in this therapeutic area in Japan.

## Supplementary Information

**Additional file 1**.** Table 1**. ICD-10 codes used for identification of cough-related diseases and exclusion criteria.** Table 2**. ATC Medication codes used to identify treatments for cough or for cough-related diseases.** Table 3**. Medication use during the post-index period by subgroup and by month.

## Data Availability

The raw data that supported the findings of this study were available from JMIRI but restrictions applied to the availability of these data, which were used under license for the current study, and so are not publicly available. The data sets generated during the current study are available from the corresponding author on reasonable requests.

## Abbreviations

ACE: Angiotensin-converting enzyme
ARNI: Allergic rhinitis / nasal inflammation
CA: Central antitussive
CAID: Chronic airway inflammatory disease
ICS: Inhaled corticosterois
LABA: Long-acting beta-agonist
GERD: Gastro-esophageal reflux disease
GI: Gastrointestinal
JRS: Japanese resoiratory society
JMIRI: Japan medical information research institute inc.
ICD-10: International classification of diseases, 10th edition
ATC: Anatomical, therapeutic and chemical class

## References

[1] Irwin RS, Baumann MH, Bolser DC, Boulet LP, Braman SS, Brightling CE, Brown KK, Canning BJ, Chang AB, Dicpinigaitis PV (2006). Diagnosis and management of cough executive summary: ACCP evidence-based clinical practice guidelines. Chest.

[2] French CL, Irwin RS, Curley FJ, Krikorian CJ (1998). Impact of chronic cough on quality of life. Arch Intern Med.

[3] Zeiger RS, Schatz M, Butler RK, Weaver JP, Bali V, Chen W (2020). Burden of specialist-diagnosed chronic cough in adults. J Allergy Clin Immunol Pract.

[4] Kubo T, Tobe K, Okuyama K, Kikuchi M, Chen Y, Schelfhout J, Abe M, Tokita S (2021). Disease burden and quality of life of patients with chronic cough in Japan: a population-based cross-sectional survey. BMJ Open Respir Res.

[5] Irwin RS, French CL, Chang AB, Altman KW (2018). Classification of cough as a symptom in adults and management algorithms: CHEST guideline and expert panel report. Chest.

[6] Morice AH, Millqvist E, Bieksiene K, Birring SS, Dicpinigaitis P, Domingo Ribas C, Hilton Boon M, Kantar A, Lai K, McGarvey L (2020). ERS guidelines on the diagnosis and treatment of chronic cough in adults and children. Eur Respir J.

[7] Lee KK, Davenport PW, Smith JA, Irwin RS, McGarvey L, Mazzone SB, Birring SS (2021). Global physiology and pathophysiology of cough: part 1: cough phenomenology - CHEST guideline and expert panel report. Chest.

[8] Chung KF, Canning B, McGarvey L (2015). Eight international london cough symposium 2014: cough hypersensitivity syndrome as the basis for chronic cough. Pulm Pharmacol Ther.

[9] Morice AH, Millqvist E, Belvisi MG, Bieksiene K, Birring SS, Chung KF, Dal Negro RW, Dicpinigaitis P, Kantar A, McGarvey LP (2014). Expert opinion on the cough hypersensitivity syndrome in respiratory medicine. Eur Respir J.

[10] Morice A, Dicpinigaitis P, McGarvey L, Birring SS (2021). Chronic cough: new insights and future prospects. Eur Respir Rev.

[11] Arinze JT, de Roos EW, Karimi L, Verhamme KMC, Stricker BH, Brusselle GG (2020). Prevalence and incidence of, and risk factors for chronic cough in the adult population: the Rotterdam study. ERJ Open Res.

[12] Fujimura M (2012). Frequency of persistent cough and trends in seeking medical care and treatment-results of an internet survey. Allergol Int.

[13] Desalu OO, Salami AK, Fawibe AE (2011). Prevalence of cough among adults in an urban community in Nigeria. West Afr J Med.

[14] Ford AC, Forman D, Moayyedi P, Morice AH (2006). Cough in the community: a cross sectional survey and the relationship to gastrointestinal symptoms. Thorax.

[15] Tobe K, Kubo T, Okuyama K, Kikuchi M, Chen Y, Schelfhout J, Abe M, Tokita S (2021). Web-based survey to evaluate the prevalence of chronic and subacute cough and patient characteristics in Japan. BMJ Open Respir Res.

[16] Meltzer EO, Zeiger RS, Dicpinigaitis P, Bernstein JA, Oppenheimer JJ, Way NA, Li VW, Boggs R, Doane MJ, Urdaneta E (2021). Prevalence and burden of chronic cough in the United States. J Allergy Clin Immunol Pract.

[17] Çolak Y, Nordestgaard BG, Laursen LC, Afzal S, Lange P, Dahl M (2017). Risk factors for chronic cough among 14,669 individuals from the general population. Chest.

[18] Mcgarvey L, Morice A, Way N, Li V, Weaver J, Doshi I, Urdaneta E, Boggs R (2019). Prevalence of chronic cough, patient characteristics and health outcomes among UK adults. Eur Resp J.

[19] Mukae H, Kaneko T, Obase Y, Shinkai M, Katsunuma T, Takeyama K, Terada J, Niimi A, Matsuse H, Yatera K et al: The Japanese respiratory society guidelines for the management of cough and sputum (digest edition). Respir Investig 2021, 59(3):270–290. 10.1016/j.resinv.2021.01.007 33642231

[20] Fujimura M, Abo M, Ogawa H, Nishi K, Kibe Y, Hirose T, Nakatsumi Y, Iwasa K (2005). Importance of atopic cough, cough variant asthma and sinobronchial syndrome as causes of chronic cough in the Hokuriku area of Japan. Respirology.

[21] Kanemitsu Y, Kurokawa R, Takeda N, Takemura M, Fukumitsu K, Asano T, Yap J, Suzuki M, Fukuda S, Ohkubo H (2019). Clinical impact of gastroesophageal reflux disease in patients with subacute/chronic cough. Allergol Int.

[22] Osokogu OU, Khan J, Nakato S, Weibel D, de Ridder M, Sturkenboom M, Verhamme K (2018). Choice of time period to identify confounders for propensity score matching, affected the estimate: a retrospective cohort study of drug effectiveness in asthmatic children. J Clin Epidemiol.

[23] Wu Q, Shen W, Cheng H, Zhou X (2014). Long-term macrolides for non-cystic fibrosis bronchiectasis: a systematic review and meta-analysis. Respirology.

[24] Song WJ, Chang YS, Faruqi S, Kim JY, Kang MG, Kim S, Jo EJ, Kim MH, Plevkova J, Park HW (2015). The global epidemiology of chronic cough in adults: a systematic review and meta-analysis. Eur Respir J.

[25] Zeiger RS, Xie F, Schatz M, Hong BD, Weaver JP, Bali V, Schelfhout J, Chen W (2020). Prevalence and characteristics of chronic cough in adults identified by administrative data. Perm J.

