# Supplementary material for: Clinical characteristics and drug utilisation patterns in patients with chronic cough: a retrospective cohort study using a Japanese claims database
Source: BMC Pulm Med. 2022 Nov 21;22:429. doi: 10.1186/s12890-022-02180-y (PMC9677640; doi:10.1186/s12890-022-02180-y)
Supplement: Supplementary file 1 — Additional file 1. Table 1. ICD-10 codes used for identification of cough-related diseases and exclusion criteria. Table 2. ATC Medication codes used to identify treatments for cough or for cough-related diseases. Table 3. Medication use during the post-index period by subgroup and by month. [file 12890_2022_2180_MOESM1_ESM.docx]

**Clinical characteristics and drug utilisation patterns in patients with chronic cough: a retrospective cohort study using a Japanese claims database**

**Yoko Arai,^1^ Kotoba Okuyama,^1^ Yoshie Onishi,^2^ Jonathan Schelfhout,^3^ Shigeru Tokita^1^ and Takekazu Kubo^1^**

**Additional Material**

**Additional Material Table 1.** ICD-10 codes used for identification of cough-related diseases and exclusion criteria.

**COUGH-RELATED DISEASES**

| **Category** | **Standard disease name** | **ICD-10 code** | **Disease code** |
| --- | --- | --- | --- |
| **Chronic airway inflammatory disease** | Smoker's Bronchitis | J410 | 8832233 |
|  | Simple Chronic Bronchitis | J410 | 8837134 |
|  | Mucopurulent Chronic Bronchitis | J411 | 8838670 |
|  | Chronic Purulent Bronchitis | J411 | 8840311 |
|  | Chronic Bronchitis | J42 | 4919002 |
|  | Chronic Tracheitis | J42 | 8840330 |
|  | Chronic Tracheobronchitis | J42 | 8840331 |
|  | Chronic Bronchorrhea | J42 | 8840333 |
|  | Acute Exacerbation of Chronic Obstructive Pulmonary Disease | J441 | 8849212 |
|  | Diffuse Panbronchiolitis | J448 | 4912001 |
|  | Obstructive Bronchitis | J448 | 4912003 |
|  | Obliterative Bronchiolitis | J448 | 4912004 |
|  | Emphysematous Chronic Obstructive Pulmonary Disease | J448 | 8849097 |
|  | Non-emphysematous Chronic Obstructive Pulmonary Disease | J448 | 8849192 |
|  | Chronic Obstructive Pulmonary Disease | J449 | 8840399 |
| **Emphysema** | Unilateral Pulmonary Emphysema | J430 | 8830557 |
|  | MacLeod Syndrome | J430 | 8840234 |
|  | Panlobular Emphysema | J431 | 8839011 |
|  | Centrilobular Emphysema | J432 | 8837582 |
|  | Pulmonary Emphysema | J439 | 4920017 |
|  | Chronic Emphysema | J439 | 4920020 |
|  | Emphysematous Pulmonary Cyst | J439 | 8832218 |
|  | Giant Emphysematous Bulla | J439 | 8832694 |
|  | Alveolar Emphysema | J439 | 8838902 |
|  | Bullous Emphysema | J439 | 8839846 |
|  | Obstructive Emphysema | J439 | 8839950 |
|  | Lung Interlobular Emphysema | J439 | 8834830 |
| **Asthma** | Atopic Asthma | J450 | 4930001 |
|  | Extrinsic Asthma | J450 | 4930002 |
|  | Allergic Bronchitis | J450 | 4939003 |
|  | Allergic Asthma | J450 | 4939004 |
|  | Psychogenic Asthma | J451 | 4939016 |
|  | Nonatopic Asthma | J451 | 4939039 |
|  | Infectious Asthma | J451 | 8831609 |
|  | Mixed Asthma | J458 | 8833884 |
|  | Bronchial Asthma | J459 | 4939008 |
|  | Asthmatic Bronchitis | J459 | 4939022 |
|  | Intractable Asthma | J459 | 4939037 |
|  | Nocturnal Asthma | J459 | 4939038 |
|  | Exercise-induced Asthma | J459 | 8841641 |
|  | Asthma | J459 | 4939020 |
|  | Chronic Asthmatic Bronchitis | J459 | 8840365 |
|  | Steroid-dependent Asthma | J459 | 8844994 |
|  | Status Asthmaticus | J46 | 4939010 |
|  | Asthmatic Attack | J46 | 4939012 |
| **Paranasal sinusitis** | Odontogenic Maxillary Sinusitis | J320 | 4730002 |
|  | Maxillary Sinusitis | J320 | 4730004 |
|  | Oroantral Fistula | J320 | 8833399 |
|  | Frontal Sinusitis | J321 | 4731001 |
|  | Ethmoiditis | J322 | 4732002 |
|  | Sphenoid Sinusitis | J323 | 4733001 |
|  | Pansinusitis | J324 | 8839031 |
|  | Eosinophilic Sinusitis | J328 | 8845156 |
|  | Caseous Sinusitis | J329 | 0128003 |
|  | Odontogenic Sinusitis | J329 | 4619007 |
|  | Sinusitis | J329 | 4739007 |
|  | Chronic Sinusitis | J329 | 4739014 |
|  | Chronic Sinusitis Acute Exacerbation | J329 | 4739015 |
|  | Purulent Sinusitis | J329 | 8831418 |
|  | Sinobronchial Syndrome | J398 | 4738006 |
|  | Chronic Infection of Upper Respiratory Tract | J398 | 4769001 |
|  | Tracheomalacia | J398 | 5191021 |
|  | Tracheostenosis | J398 | 8832150 |
|  | Tracheocele | J398 | 8832190 |
|  | Nasopharyngeal Closure Insufficiency | J398 | 8839447 |
|  | Nasopharyngeal Closure Insufficiency | J398 | 8849194 |
|  | Sinobronchitis Syndrome | J398 | 4738005 |
|  | Tracheoesophageal Stenosis | J398 | 5303007 |
| **Postnasal drip** | Postnasal Drip | J348 | 8846279 |
|  | Dry Anterior Rhinitis | J348 | 4720006 |
|  | Intranasal Erosion | J348 | 4781018 |
|  | Perforation of Nasal Septum | J348 | 4781026 |
|  | Rhinalgia | J348 | 4781031 |
|  | Intranasal Adhesion | J348 | 4781032 |
|  | Rhinorrhoea | J348 | 4781041 |
|  | Nasal Hydrorrhea | J348 | 4781057 |
|  | Purulent Nasal Obstruction | J348 | 4781058 |
|  | Mucinous Rhinorrhoea | J348 | 4781059 |
|  | Bloody Rhinorrhoea | J348 | 4781060 |
|  | Choanal Stenosis | J348 | 7480006 |
|  | Nasal Vestibulitis | J348 | 8839481 |
|  | Nasal Vestibule Erosion | J348 | 8839484 |
|  | Nasal Septum Perichondritis | J348 | 4781028 |
|  | Rhinolith | J348 | 8838973 |
| **Vocal cord dysfunction** | Laryngeal Paralysis | J380 | 4783004 |
|  | Vocal Cord Paralysis | J380 | 4783010 |
|  | Abductor Paralysis | J380 | 4783019 |
|  | Vocal Cord Paresis | J380 | 8835926 |
|  | Vocal Cord Pseudoparalysis | J380 | 4783002 |
|  | Polypoid Vocal Fold | J381 | 4784001 |
|  | Vocal Cord Polyp | J381 | 4784003 |
|  | Vocal Chorditis | J382 | 4785009 |
|  | Edematous Chorditis | J382 | 4785016 |
|  | Vocal Nodule | J382 | 8835923 |
|  | Sulcus Vocalis | J383 | 4785012 |
|  | Vocal Cord Cicatrization | J383 | 4785025 |
|  | Atrophic Vocal Chorditis | J383 | 8830445 |
|  | Vocal Fold Cyst | J383 | 8842303 |
|  | Spasmodic Dysphonia | J383 | 8843703 |
|  | Vocal Cord Atrophy | J383 | 8843741 |
|  | Vocal Cord Dysfunction | J383 | 8844888 |
|  | Laryngeal Edema | J384 | 4786002 |
|  | Glottis Edema | J384 | 4786005 |
|  | Subglottic Edema | J384 | 8835960 |
|  | Laryngospasm | J385 | 4787021 |
|  | Spastic Laryngotracheitis | J385 | 8833026 |
|  | Laryngostenosis | J386 | 8833612 |
|  | Laryngeal Obstruction | J386 | 4787019 |
|  | Allergic Laryngitis | J387 | 4640012 |
|  | Epiglottic Cyst | J387 | 8833607 |
|  | Laryngeal Perichondritis | J387 | 8833623 |
|  | Phlegmonous Laryngitis | J387 | 8833642 |
|  | Throat Larynx Reflux | J387 | 8842672 |
|  | Laryngoptosis | J387 | 8833601 |
|  | Epiglottic Perichondritis | J387 | 8833606 |
|  | Larynx Hypofunction | J387 | 8833611 |
| **Allergic rhinitis / Nasal inflammation** | Vasomotor Rhinitis | J300 | 8833084 |
|  | Pollinosis | J301 | 4770002 |
|  | Allergic Rhinoconjunctivitis | J301 | 8845847 |
|  | Poaceous Pollinosis | J301 | 8848025 |
|  | Orchard Grass Pollinosis | J301 | 8848033 |
|  | Japanese Cedar Pollinosis | J301 | 8848069 |
|  | Japanese Cypress Pollinosis | J301 | 8848097 |
|  | Ragweed Pollinosis | J301 | 8848099 |
|  | Seasonal Allergic Rhinitis | J302 | 4779016 |
|  | Perennial Allergic Rhinitis | J303 | 8837835 |
|  | Allergic Rhinitis | J304 | 4779004 |
|  | Allergic Sinusitis | J304 | 4779015 |
|  | Allergic Nasopharyngitis | J304 | 8830398 |
|  | Nasal Allergy | J304 | 4779013 |
|  | Rhinitis | J310 | 4781015 |
| **Chronic rhinitis** | Atrophic Rhinitis | J310 | 4720003 |
|  | Suppurative Rhinitis | J310 | 4720004 |
|  | Dry Rhinitis | J310 | 4720005 |
|  | Ozena | J310 | 4720008 |
|  | Chronic Rhinitis | J310 | 4720021 |
|  | Congestive Rhinitis | J310 | 8830797 |
|  | Catarrhal Rhinitis | J310 | 8831368 |
|  | Hypertrophic Rhinitis | J310 | 8839217 |
|  | Obliterative Rhinitis | J310 | 8839951 |
|  | Eosinophilic Rhinitis | J310 | 8844050 |
|  | Rhinorrhea | J310 | 8844817 |
|  | Nasopharyngeal Atrophy | J311 | 8839439 |
|  | Chronic Purulent Nasopharyngitis | J311 | 8840314 |
|  | Chronic Nasopharyngitis | J311 | 8840390 |
|  | Chronic Pharyngitis | J312 | 4721007 |
|  | Atrophic Pharyngitis | J312 | 8830439 |
|  | Granular Pharyngitis | J312 | 8831463 |
|  | Dry Pharyngitis | J312 | 8831639 |
|  | Chronic Pharyngeal Catarrh | J312 | 8840303 |
|  | Spotted Sore Throat | J312 | 8841264 |
| **Nasal polyp** | Nasal Polyp | J330 | 4710002 |
|  | Choanal Polyp | J330 | 8833665 |
|  | Antral Polyp | J338 | 8835400 |
|  | Sphenoidal Sinus Polyp | J338 | 8837711 |
|  | Sinus Polyp | J338 | 8839721 |
|  | Ethmoidal Sinus Polyp | J338 | 8834283 |
|  | Antro Choanal Polyp | J338 | 8846698 |
|  | Bleeding Polyp | J339 | 4709003 |
|  | Nasal Polyp | J339 | 8843493 |
| **Gastro-oesophageal reflux disease (including non-erosive)** | Reflux Esophagitis | K210 | 5301002 |
|  | Intractable Regurgitant Esophagitis | K210 | 8838366 |
|  | Refractory Reflux Esophagitis Requiring Maintenance Therapy | K210 | 8843825 |
|  | Postoperative Reflux Esophagitis | K210 | 8844239 |
|  | Postoperative Refractory Reflux Esophagitis Requiring Maintenance Therapy | K210 | 8845215 |
|  | Postoperative Intractable Regurgitant Esophagitis | K210 | 8845322 |
|  | Gastro-oesophageal Reflux Disease | K219 | 8842668 |
|  | Non-erosive Reflux Disease | K219 | 8843104 |

**Exclusion Criteria**

| **Disease** | **ICD-10 description** | **ICD-10 code** |
| --- | --- | --- |
| **Malignant neoplasms** | Malignant neoplasms | C00-C97 |
|  | In situ neoplasms | D00-D09 |
| **Interstitial lung disease** | Other respiratory diseases principally affecting the interstitium (except eosinophilic bronchitis) | J80, J81, J83, J84 |
|  | Drug-induced interstitial lung disorders | J70.2-J70.4 |
|  | Interstitial emphysema | J98.2 |
| **Cystic fibrosis** | Cystic fibrosis | E84 |
| **Pulmonary fibrosis** | Other interstitial pulmonary diseases with fibrosis | J84.1 |
|  | Chronic and other pulmonary manifestations due to radiation - fibrosis of lung following radiation | J70.1 |
|  | Silicosis | J62.8 |

**Additional Material Table 2.** ATC Medication codes used to identify treatments for cough or for cough-related diseases.

| **Category** | **Class/Type** | **Drug brand name (examples)** | **Indication** |
| --- | --- | --- | --- |
| Central antitussives | Narcotic | Codeine phosphate, Dihydrocodeine phosphate | Used as non-specific treatments |
|  | Non-narcotic | *Asverin®, Medicon®, Astomin®, Resplen®, Hustazol®, Flaveric®, Coldrin®* |  |
| Bronchodilators | Theophylline derivatives | *Theodur®, Theolong®, Slo-bid®, Uniphyl®,*  *Unicon®* | Specifically used in cough- variant asthma |
|  | β_2_ agonists (LABA alone or SABA) | *Sultanol®, Meptin®, Hokunalin tapes®, Spiropent®, Serevent®* |  |
|  | Anticholinergics | *Atrovent®, Spiriva® Respimat®* |  |
| Corticosteroids | Oral steroids  Inhaled steroids (ICS alone) | *Predonine®, Rinderon®, Qvar®, Flutide®, Pulmicort®, Alvesco®, Asmanex®* | Specifically used in cough variant asthma and atopic cough |
| ICS and LABA combination |  | *Adoair®, Symbicort®, Flutiform®, Relvar®*  *(combination of one LABA device, e.g. Spiropent® and one ICS device, e.g. Pulmicort®)* | Specifically used in cough variant asthma |
| Antimicrobials | Respiratory quinolones | *Cravit®, Ozex®, Avelox®, Geninax®, Gracevit®* | Specifically used in mycoplasma, *Chlamydia*, and whooping cough |
|  | 14- and 15-member ring macrolides | *Erythrocin®, Clarith®, Klaricid®, Rulid®, Zithromac®* | The same as above as well as SBS |
|  | Other antimicrobials | (Omitted) | Used in specific respiratory infections |
| Expectorant |  | *Mucofilin®, Bisolvon®, Mucodyne®, Cleanal®, Spelear®, Mucosolvan* | Specifically used in each type of wet cough |
| Herbal medicine |  | *Bakumondo-to, Saiboku-to, Xiao-qing-long tang, Seihai-to, Jiinkoka-to, Hangekoboku-to* | Used as non-specific treatments |
| Antiallergic agents | Histamine H1 receptor antagonists | *Azeptin®, Celtect®, Allegra®, Alesion®, Ebastel®, Zyrtec®, Xyzal®, Talion®, Remicut®, Daren®, Allelock®, Claritin®, Desalex®, Bilanoa®, Rupafin®* | Specifically used in atopic cough, laryngeal allergy (chronic), and postinfectious cough (non-specific) |
|  | Leukotriene receptor antagonists | *Onon®, Singulair®, Kipres®* | Specifically used in cough variant asthma |
|  | Thromboxane inhibitors | *Vega®, Domenan®, Bronica®, Baynas®* |  |
|  | Th2 cytokine inhibitors | *IPD®* |  |
| Drugs for peptic ulcer treatment | Histamine H_2_ receptor antagonists | *Gaster®, Zantac®, Tagamet®, Altat®, Acinon®, Protecadin®, Nizatoric®* | Specifically used in cough caused by GERD |
|  | Proton pump inhibitors | *Omepral®, Omeprazon®, Takepron®, Pariet®, Nexium®, Takecab®* |  |
| Drugs for improvement of gastrointestinal motility function |  | *Primperan®, Nauzelin®, Ganaton®, Cerekinon®, Gasmotin®, Acofide®* |  |

ICS: inhaled corticosteroid; GERD: gastro-oesophageal reflux disease; LABA: long-acting beta-agonist; SABA: long-acting beta-agonist; SBS: sinobronchial syndrome.

**Additional Material Table 3.** Medication use during the post-index period by subgroup and by month.

|  | **Population ‘All’** | | **Population 1** | | **Population 2** | | **Other coughs** | | **Cough-variant asthma** | | **Atopic cough / allergic cough** | |
| --- | --- | --- | --- | --- | --- | --- | --- | --- | --- | --- | --- | --- |
| **Month 1** | **N = 6,038** | **(%)** | **N = 3,500** | **(%)** | **N = 2,538** | **(%)** | **N = 1,444** | **(%)** | **N = 1,026** | **(%)** | **N = 105** | **(%)** |
| Any drugs of interest | 2015 |  | 1016 |  | 999 |  | 575 |  | 403 |  | 40 |  |
| Central antitussives (alone*) | 27 | 1.3 | 18 | 1.8 | 9 | 0.9 | 2 | 0.3 | 5 | 1.2 | 1 | 2.5 |
| Bronchodilators (without CA) | 127 | 6.3 | 54 | 5.3 | 73 | 7.3 | 44 | 7.7 | 32 | 7.9 | 1 | 2.5 |
| + Central antitussives | 80 | 4.0 | 54 | 5.3 | 26 | 2.6 | 18 | 3.1 | 9 | 2.2 | 2 | 5 |
| Corticosteroids (without CA) | 412 | 20.4 | 188 | 18.5 | 224 | 22.4 | 132 | 23.0 | 90 | 22.3 | 4 | 10 |
| + Central antitussives | 88 | 4.4 | 44 | 4.3 | 44 | 4.4 | 33 | 5.7 | 12 | 3.0 | 0 | 0 |
| ICS and LABA combination (without CA) | 440 | 21.8 | 220 | 21.7 | 220 | 22.0 | 91 | 15.8 | 123 | 30.5 | 11 | 27.5 |
| + Central antitussives | 54 | 2.7 | 37 | 3.6 | 17 | 1.7 | 6 | 1.0 | 11 | 2.7 | 1 | 2.5 |
| Antimicrobials (without CA) | 364 | 18.1 | 200 | 19.7 | 164 | 16.4 | 108 | 18.8 | 61 | 15.1 | 5 | 12.5 |
| + Central antitussives | 67 | 3.3 | 43 | 4.2 | 24 | 2.4 | 14 | 2.4 | 9 | 2.2 | 1 | 2.5 |
| Expectorant (without CA) | 596 | 29.6 | 331 | 32.6 | 265 | 26.5 | 161 | 28.0 | 103 | 25.6 | 8 | 20 |
| + Central antitussives | 68 | 3.4 | 41 | 4.0 | 27 | 2.7 | 20 | 3.5 | 7 | 1.7 | 0 | 0 |
| Herbal medicine (without CA) | 312 | 15.5 | 145 | 14.3 | 167 | 16.7 | 132 | 23.0 | 36 | 8.9 | 4 | 10 |
| + Central antitussives | 11 | 0.5 | 8 | 0.8 | 3 | 0.3 | 2 | 0.3 | 1 | 0.2 | 0 | 0 |
| Antiallergic agents (without CA) | 1059 | 52.6 | 516 | 50.8 | 543 | 54.4 | 296 | 51.5 | 226 | 56.1 | 27 | 67.5 |
| + Central antitussives | 25 | 1.2 | 13 | 1.3 | 12 | 1.2 | 9 | 1.6 | 2 | 0.5 | 1 | 2.5 |
| Drugs for peptic ulcer treatment (without CA) | 335 | 16.6 | 163 | 16.0 | 172 | 17.2 | 97 | 16.9 | 69 | 17.1 | 9 | 22.5 |
| + Central antitussives | 4 | 0.2 | 2 | 0.2 | 2 | 0.2 | 1 | 0.2 | 1 | 0.2 | 0 | 0 |
| Drugs for improvement of gastrointestinal motility function (without CA) | 137 | 6.8 | 71 | 7.0 | 66 | 6.6 | 44 | 7.7 | 24 | 6.0 | 5 | 12.5 |
| + Central antitussives | 1 | 0.0 | 0 | 0 | 1 | 0.1 | 1 | 0.2 | 0 | 0 | 0 | 0 |
| **Month 2** | **N = 6,038** |  | **N = 3,500** |  | **N = 2,538** |  | **N = 1,444** |  | **N = 1,026** |  | **N = 105** |  |
| Any drugs of interest | 1833 |  | 901 |  | 932 |  | 543 |  | 368 |  | 41 |  |
| Central antitussives (alone*) | 27 | 1.5 | 19 | 2.1 | 8 | 0.9 | 5 | 0.9 | 3 | 0.8 | 0 | 0.0 |
| Bronchodilators (without CA) | 105 | 5.7 | 45 | 5.0 | 60 | 6.4 | 39 | 7.2 | 22 | 6.0 | 1 | 2.4 |
| + Central antitussives | 67 | 3.7 | 29 | 3.2 | 38 | 4.1 | 28 | 5.2 | 10 | 2.7 | 1 | 2.4 |
| Corticosteroids (without CA) | 373 | 20.3 | 161 | 17.9 | 212 | 22.7 | 143 | 26.3 | 69 | 18.8 | 3 | 7.3 |
| + Central antitussives | 46 | 2.5 | 23 | 2.6 | 23 | 2.5 | 10 | 1.8 | 12 | 3.3 | 1 | 2.4 |
| ICS and LABA combination (without CA) | 340 | 18.5 | 146 | 16.2 | 194 | 20.8 | 92 | 16.9 | 96 | 26.1 | 10 | 24.4 |
| + Central antitussives | 58 | 3.2 | 29 | 3.2 | 29 | 3.1 | 12 | 2.2 | 14 | 3.8 | 3 | 7.3 |
| Antimicrobials (without CA) | 314 | 17.1 | 173 | 19.2 | 141 | 15.1 | 89 | 16.4 | 50 | 13.6 | 7 | 17.1 |
| + Central antitussives | 76 | 4.1 | 48 | 5.3 | 28 | 3.0 | 14 | 2.6 | 13 | 3.5 | 1 | 2.4 |
| Expectorant (without CA) | 515 | 28.1 | 274 | 30.4 | 241 | 25.9 | 137 | 25.2 | 96 | 26.1 | 12 | 29.3 |
| + Central antitussives | 69 | 3.8 | 35 | 3.9 | 34 | 3.6 | 19 | 3.5 | 14 | 3.8 | 2 | 4.9 |
| Herbal medicine (without CA) | 262 | 14.3 | 116 | 12.9 | 146 | 15.7 | 114 | 21.0 | 29 | 7.9 | 4 | 9.8 |
| + Central antitussives | 4 | 0.2 | 0 | 0.0 | 4 | 0.4 | 3 | 0.6 | 1 | 0.3 | 0 | 0.0 |
| Antiallergic agents (without CA) | 958 | 52.3 | 447 | 49.6 | 511 | 54.8 | 289 | 53.2 | 203 | 55.2 | 30 | 73.2 |
| + Central antitussives | 14 | 0.8 | 7 | 0.8 | 7 | 0.8 | 6 | 1.1 | 1 | 0.3 | 0 | 0.0 |
| Drugs for peptic ulcer treatment (without CA) | 358 | 19.5 | 183 | 20.3 | 175 | 18.8 | 96 | 17.7 | 70 | 19.0 | 8 | 19.5 |
| + Central antitussives | 5 | 0.3 | 2 | 0.2 | 3 | 0.3 | 3 | 0.6 | 0 | 0.0 | 0 | 0.0 |
| Drugs for improvement of gastrointestinal motility function (without CA) | 124 | 6.8 | 61 | 6.8 | 63 | 6.8 | 42 | 7.7 | 21 | 5.7 | 4 | 9.8 |
| + Central antitussives | 1 | 0.1 | 1 | 0.1 | 0 | 0.0 | 0 | 0.0 | 0 | 0.0 | 0 | 0.0 |
| **Month 3** | **N = 6,038** |  | **N = 3,500** |  | **N = 2,538** |  | **N = 1,444** |  | **N = 1,026** |  | **N = 105** |  |
| Any drugs of interest | 1693 |  | 827 |  | 866 |  | 499 |  | 339 |  | 38 |  |
| Central antitussives (alone*) | 19 | 1.1 | 10 | 1.2 | 9 | 1.0 | 7 | 1.4 | 2 | 0.6 | 0 | 0.0 |
| Bronchodilators (without CA) | 106 | 6.3 | 55 | 6.7 | 51 | 5.9 | 23 | 4.6 | 28 | 8.3 | 2 | 5.3 |
| + Central antitussives | 56 | 3.3 | 28 | 3.4 | 28 | 3.2 | 119 | 23.8 | 74 | 21.8 | 6 | 15.8 |
| Corticosteroids (without CA) | 366 | 21.6 | 169 | 20.4 | 197 | 22.7 | 20 | 4.0 | 9 | 2.7 | 1 | 2.6 |
| + Central antitussives | 71 | 4.2 | 36 | 4.4 | 35 | 4.0 | 22 | 4.4 | 11 | 3.2 | 2 | 5.3 |
| ICS and LABA combination (without CA) | 313 | 18.5 | 135 | 16.3 | 178 | 20.6 | 78 | 15.6 | 96 | 28.3 | 7 | 18.4 |
| + Central antitussives | 38 | 2.2 | 19 | 2.3 | 19 | 2.2 | 10 | 2.0 | 7 | 2.1 | 3 | 7.9 |
| Antimicrobials (without CA) | 332 | 19.6 | 192 | 23.2 | 140 | 16.2 | 85 | 17.0 | 49 | 14.5 | 8 | 21.1 |
| + Central antitussives | 79 | 4.7 | 44 | 5.3 | 35 | 4.0 | 24 | 4.8 | 10 | 2.9 | 2 | 5.3 |
| Expectorant (without CA) | 499 | 29.5 | 276 | 33.4 | 223 | 25.8 | 122 | 24.4 | 95 | 28.0 | 10 | 26.3 |
| + Central antitussives | 64 | 3.8 | 30 | 3.6 | 34 | 3.9 | 22 | 4.4 | 13 | 3.8 | 0 | 0.0 |
| Herbal medicine (without CA) | 238 | 14.1 | 106 | 12.8 | 132 | 15.2 | 109 | 21.8 | 24 | 7.1 | 2 | 5.3 |
| + Central antitussives | 3 | 0.2 | 2 | 0.2 | 1 | 0.1 | 1 | 0.2 | 0 | 0.0 | 0 | 0.0 |
| Antiallergic agents (without CA) | 913 | 53.9 | 439 | 53.1 | 474 | 54.7 | 259 | 51.9 | 189 | 55.8 | 28 | 73.7 |
| + Central antitussives | 10 | 0.6 | 2 | 0.2 | 8 | 0.9 | 5 | 1.0 | 3 | 0.9 | 0 | 0.0 |
| Drugs for peptic ulcer treatment (without CA) | 297 | 17.5 | 135 | 16.3 | 162 | 18.7 | 84 | 16.8 | 65 | 19.2 | 13 | 34.2 |
| + Central antitussives | 3 | 0.2 | 3 | 0.4 | 0 | 0.0 | 0 | 0.0 | 0 | 0.0 | 0 | 0.0 |
| Drugs for improvement of gastrointestinal motility function (without CA) | 106 | 6.3 | 51 | 6.2 | 55 | 6.4 | 37 | 7.4 | 18 | 5.3 | 2 | 5.3 |
| + Central antitussives | 2 | 0.1 | 1 | 0.1 | 1 | 0.1 | 1 | 0.2 | 0 | 0.0 | 0 | 0.0 |
| **Month 6** | **N = 6,038** |  | **N = 3,500** |  | **N = 2,538** |  | **N = 1,444** |  | **N = 1,026** |  | **N = 105** |  |
| Any drugs of interest | 1491 |  | 695 |  | 796 |  | 458 |  | 316 |  | 37 |  |
| Central antitussives (alone*) | 22 | 1.5 | 16 | 2.3 | 6 | 0.8 | 4 | 0.9 | 1 | 0.3 | 1 | 2.7 |
| Bronchodilators (without CA) | 83 | 5.6 | 32 | 4.6 | 51 | 6.4 | 30 | 6.6 | 21 | 6.6 | 2 | 5.4 |
| + Central antitussives | 66 | 4.4 | 30 | 4.3 | 36 | 4.5 | 21 | 4.6 | 15 | 4.7 | 0 | 0.0 |
| Corticosteroids (without CA) | 322 | 21.6 | 143 | 20.6 | 179 | 22.5 | 108 | 23.6 | 65 | 20.6 | 8 | 21.6 |
| + Central antitussives | 48 | 3.2 | 22 | 3.2 | 26 | 3.3 | 15 | 3.3 | 8 | 2.5 | 4 | 10.8 |
| ICS and LABA combination (without CA) | 279 | 18.7 | 101 | 14.5 | 178 | 22.4 | 83 | 18.1 | 86 | 27.2 | 10 | 27.0 |
| + Central antitussives | 31 | 2.1 | 16 | 2.3 | 15 | 1.9 | 6 | 1.3 | 7 | 2.2 | 2 | 5.4 |
| Antimicrobials (without CA) | 293 | 19.7 | 156 | 22.4 | 137 | 17.2 | 76 | 16.6 | 51 | 16.1 | 10 | 27.0 |
| + Central antitussives | 72 | 4.8 | 44 | 6.3 | 28 | 3.5 | 20 | 4.4 | 11 | 3.5 | 0 | 0.0 |
| Expectorant (without CA) | 452 | 30.3 | 236 | 34.0 | 216 | 27.1 | 129 | 28.2 | 81 | 25.6 | 12 | 32.4 |
| + Central antitussives | 57 | 3.8 | 29 | 4.2 | 28 | 3.5 | 13 | 2.8 | 16 | 5.1 | 0 | 0.0 |
| Herbal medicine (without CA) | 183 | 12.3 | 82 | 11.8 | 101 | 12.7 | 81 | 17.7 | 20 | 6.3 | 1 | 2.7 |
| + Central antitussives | 11 | 0.7 | 6 | 0.9 | 5 | 0.6 | 4 | 0.9 | 0 | 0.0 | 1 | 2.7 |
| Antiallergic agents (without CA) | 813 | 54.5 | 367 | 52.8 | 446 | 56.0 | 253 | 55.2 | 175 | 55.4 | 28 | 75.7 |
| + Central antitussives | 7 | 0.5 | 2 | 0.3 | 5 | 0.6 | 4 | 0.9 | 0 | 0.0 | 0 | 0.0 |
| Drugs for peptic ulcer treatment (without CA) | 270 | 18.1 | 117 | 16.8 | 153 | 19.2 | 81 | 17.7 | 66 | 20.9 | 9 | 24.3 |
| + Central antitussives | 5 | 0.3 | 3 | 0.4 | 2 | 0.3 | 1 | 0.2 | 0 | 0.0 | 1 | 2.7 |
| Drugs for improvement of gastrointestinal motility function (without CA) | 114 | 7.6 | 53 | 7.6 | 61 | 7.7 | 38 | 8.3 | 21 | 6.6 | 4 | 10.8 |
| + Central antitussives | 2 | 0.1 | 1 | 0.1 | 1 | 0.1 | 1 | 0.2 | 0 | 0.0 | 0 | 0.0 |
| **Month 9** | **N = 6,038** |  | **N = 3,500** |  | **N = 2,538** |  | **N = 1,444** |  | **N = 1,026** |  | **N = 105** |  |
| Any drugs of interest | 1392 |  | 666 |  | 726 |  | 409 |  | 295 |  | 31 |  |
| Central antitussives (alone*) | 21 | 1.5 | 13 | 2.0 | 8 | 1.1 | 5 | 1.2 | 3 | 1.0 | 0 | 0.0 |
| Bronchodilators (without CA) | 85 | 6.1 | 36 | 5.4 | 49 | 6.7 | 26 | 6.4 | 23 | 7.8 | 2 | 6.5 |
| + Central antitussives | 59 | 4.2 | 35 | 5.3 | 24 | 3.3 | 15 | 3.7 | 10 | 3.4 | 0 | 0.0 |
| Corticosteroids (without CA) | 288 | 20.7 | 125 | 18.8 | 163 | 22.5 | 89 | 21.8 | 67 | 22.7 | 12 | 38.7 |
| + Central antitussives | 40 | 2.9 | 19 | 2.9 | 21 | 2.9 | 15 | 3.7 | 6 | 2.0 | 0 | 0.0 |
| ICS and LABA combination (without CA) | 242 | 17.4 | 92 | 13.8 | 150 | 20.7 | 65 | 15.9 | 84 | 28.5 | 7 | 22.6 |
| + Central antitussives | 36 | 2.6 | 19 | 2.9 | 17 | 2.3 | 6 | 1.5 | 11 | 3.7 | 0 | 0.0 |
| Antimicrobials (without CA) | 253 | 18.2 | 141 | 21.2 | 141 | 19.4 | 67 | 16.4 | 39 | 13.2 | 8 | 25.8 |
| + Central antitussives | 53 | 3.8 | 34 | 5.1 | 19 | 2.6 | 9 | 2.2 | 8 | 2.7 | 1 | 3.2 |
| Expectorant (without CA) | 399 | 28.7 | 206 | 30.9 | 193 | 26.6 | 106 | 25.9 | 79 | 26.8 | 9 | 29.0 |
| + Central antitussives | 49 | 3.5 | 27 | 4.1 | 22 | 3.0 | 15 | 3.7 | 8 | 2.7 | 0 | 0.0 |
| Herbal medicine (without CA) | 161 | 11.6 | 74 | 11.1 | 87 | 12.0 | 69 | 16.9 | 20 | 6.8 | 1 | 3.2 |
| + Central antitussives | 3 | 0.2 | 1 | 0.2 | 2 | 0.3 | 2 | 0.5 | 0 | 0.0 | 0 | 0.0 |
| Antiallergic agents (without CA) | 768 | 55.2 | 354 | 53.2 | 414 | 57.0 | 229 | 56.0 | 172 | 58.3 | 23 | 74.2 |
| + Central antitussives | 14 | 1.0 | 8 | 1.2 | 6 | 0.8 | 3 | 0.7 | 1 | 0.3 | 1 | 3.2 |
| Drugs for peptic ulcer treatment (without CA) | 240 | 17.2 | 102 | 15.3 | 138 | 19.0 | 77 | 18.8 | 54 | 18.3 | 11 | 35.5 |
| + Central antitussives | 3 | 0.2 | 1 | 0.2 | 2 | 0.3 | 1 | 0.2 | 1 | 0.3 | 0 | 0.0 |
| Drugs for improvement of gastrointestinal motility function (without CA) | 115 | 8.3 | 55 | 8.3 | 60 | 8.3 | 33 | 8.1 | 22 | 7.5 | 3 | 9.7 |
| + Central antitussives | 1 | 0.1 | 0 | 0.0 | 1 | 0.1 | 1 | 0.2 | 0 | 0.0 | 0 | 0.0 |
| **Month 12** | **N = 6,038** |  | **N = 3,500** |  | **N = 2,538** |  | **N = 1,444** |  | **N = 1,026** |  | **N = 105** |  |
| Any drugs of interest | 1317 |  | 628 |  | 689 |  | 394 |  | 281 |  | 32 |  |
| Central antitussives (alone*) | 14 | 1.1 | 7 | 1.1 | 7 | 1.0 | 1 | 0.3 | 6 | 2.1 | 0 | 0.0 |
| Bronchodilators (without CA) | 83 | 6.3 | 34 | 5.4 | 49 | 7.1 | 28 | 7.1 | 21 | 7.5 | 3 | 9.4 |
| + Central antitussives | 40 | 3.0 | 23 | 3.7 | 17 | 2.5 | 8 | 2.0 | 8 | 2.8 | 0 | 0.0 |
| Corticosteroids (without CA) | 277 | 21.0 | 137 | 21.8 | 140 | 20.3 | 80 | 20.3 | 58 | 20.6 | 4 | 12.5 |
| + Central antitussives | 54 | 4.1 | 25 | 4.0 | 29 | 4.2 | 16 | 4.1 | 14 | 5.0 | 0 | 0.0 |
| ICS and LABA combination (without CA) | 224 | 17.0 | 87 | 13.9 | 137 | 19.9 | 60 | 15.2 | 74 | 26.3 | 7 | 21.9 |
| + Central antitussives | 29 | 2.2 | 18 | 2.9 | 11 | 1.6 | 6 | 1.5 | 5 | 1.8 | 0 | 0.0 |
| Antimicrobials (without CA) | 232 | 17.6 | 134 | 21.3 | 98 | 14.2 | 56 | 14.2 | 40 | 14.2 | 5 | 15.6 |
| + Central antitussives | 57 | 4.3 | 33 | 5.3 | 24 | 3.5 | 11 | 2.8 | 12 | 4.3 | 1 | 3.1 |
| Expectorant (without CA) | 379 | 28.8 | 199 | 31.7 | 180 | 26.1 | 100 | 25.4 | 77 | 27.4 | 7 | 21.9 |
| + Central antitussives | 55 | 4.2 | 31 | 4.9 | 24 | 3.5 | 13 | 3.3 | 12 | 4.3 | 3 | 9.4 |
| Herbal medicine (without CA) | 166 | 12.6 | 70 | 11.1 | 96 | 13.9 | 73 | 18.5 | 26 | 9.3 | 1 | 3.1 |
| + Central antitussives | 8 | 0.6 | 6 | 1.0 | 2 | 0.3 | 1 | 0.3 | 0 | 0.0 | 1 | 3.1 |
| Antiallergic agents (without CA) | 723 | 54.9 | 338 | 53.8 | 385 | 55.9 | 214 | 54.3 | 162 | 57.7 | 22 | 68.8 |
| + Central antitussives | 12 | 0.9 | 6 | 1.0 | 6 | 0.9 | 5 | 1.3 | 2 | 0.7 | 1 | 3.1 |
| Drugs for peptic ulcer treatment (without CA) | 225 | 17.1 | 108 | 17.2 | 117 | 17.0 | 62 | 15.7 | 48 | 17.1 | 11 | 34.4 |
| + Central antitussives | 1 | 0.1 | 0 | 0.0 | 1 | 0.1 | 1 | 0.3 | 0 | 0.0 | 0 | 0.0 |
| Drugs for improvement of gastrointestinal motility function (without CA) | 95 | 7.2 | 44 | 7.0 | 51 | 7.4 | 32 | 8.1 | 17 | 6.0 | 2 | 6.3 |
| + Central antitussives | 0 | 0 | 0 | 0.0 | 0 | 0.0 | 0 | 0.0 | 0 | 0.0 | 0 | 0.0 |

CA: central antitussive; ICS: inhaled corticosteroid; LABA: long-acting beta-agonist.
